# Pediatric Toxidrome Simulation Curriculum: Lidocaine-Induced Methemoglobinemia

**DOI:** 10.15766/mep_2374-8265.11089

**Published:** 2021-01-28

**Authors:** Chelsea Del Rosso, Anita Thomas, Nicole Hardy, Scott Connelly, Ulysses Davila, Jean Pearce, Suzan Mazor, Rebekah Burns

**Affiliations:** 1 Chief Pediatric Resident Physician, Department of Pediatrics, University of Washington School of Medicine and Seattle Children's Hospital; 2 Assistant Professor, Department of Pediatrics, Division of Emergency Medicine, University of Washington School of Medicine and Seattle Children's Hospital; 3 Pediatric Emergency Medicine Fellow, University of North Carolina at Chapel Hill School of Medicine; 4 Assistant Professor, Department of Pediatrics, University of North Carolina at Chapel Hill School of Medicine; 5 Pediatric Emergency Medicine Fellow, Medical University of South Carolina College of Medicine; 6 Assistant Professor, Department of Pediatrics, Medical College of Wisconsin; 7 Professor, Department of Pediatrics, Division of Emergency Medicine, University of Washington School of Medicine and Seattle Children's Hospital; 8 Associate Professor, Department of Pediatrics, Division of Emergency Medicine, University of Washington School of Medicine and Seattle Children's Hospital

**Keywords:** Pediatric Emergency Medicine, Toxicology, Toxicological Phenomena, Methemoglobinemia, Simulation Curriculum, Simulation, Emergency Medicine

## Abstract

**Introduction:**

Lidocaine is a common local anesthetic used during minor procedures performed on pediatric patients. A rare but toxic and life-threatening side effect of lidocaine is methemoglobinemia. It should be considered in children who are hypoxic after exposure to an oxidizing agent.

**Methods:**

We developed this simulation case for pediatric emergency medicine (PEM) fellows, but it can be adapted for interprofessional simulation. The case involved a 1-month-old male with hypoxia and resulting central cyanosis after exposure to lidocaine. The team performed an initial evaluation and intervention, collected a history, and developed a differential diagnosis for hypoxia and central cyanosis in an infant. Methemoglobinemia was confirmed by CO-oximetry. Preparatory materials, a debriefing guide, and scenario evaluation forms assisted with facilitation.

**Results:**

Fifty-six participants (including 18 PEM fellows) completed this simulation across four institutions. Participants rated the scenario on a 5-point Likert scale (1 = *strongly disagree,* 5 = *strongly agree*), finding it to be relevant to their work (median = 5) and realistic (median = 5). After participation in the simulation, learners felt confident in their ability to recognize methemoglobinemia (median = 4) and implement a plan to stabilize an infant with hypoxia (median = 4).

**Discussion:**

This simulation represents a resource for learners in the pediatric emergency department. It teaches the recognition and management of an infant with lidocaine toxicity and resultant methemoglobinemia. It uses experiential learning to teach and reinforce a systematic approach to the evaluation and management of a critically ill infant with acquired methemoglobinemia.

## Educational Objectives

After participation in this simulation session, learners will be able to:

1. Perform a primary survey of a critically ill pediatric patient.
2. Implement a plan to stabilize a hypoxic and cyanotic neonate.
3. Develop a systematic approach for the evaluation of hypoxia and central cyanosis in a pediatric patient.
4. Describe the signs and symptoms of acquired methemoglobinemia in a pediatric patient.
5. Manage a pediatric patient with acquired methemoglobinemia.
6. Demonstrate teamwork and communication skills in a resuscitation setting.

## Introduction

Methemoglobinemia is a rare but life-threatening disorder and can present at any age after exposure to an oxidizing agent. It occurs when hemoglobin becomes altered, causing the irreversible binding of oxygen through oxidation of ferrous ions associated with heme to the ferric state. The ferric ions of heme (Fe+++) cannot dissociate from oxygen, causing a left shift in the oxyhemoglobin dissociation curve and subsequent hypoxia.^1^ The impaired oxygen delivery to the tissues causes central cyanosis even with typical supportive measures to improve tissue oxygenation.^1^ The resultant hypoxia may lead to respiratory failure, end-organ damage, and death in severe cases.^1^

Methemoglobinemia develops as the result of a congenital condition or toxin exposure. Congenital methemoglobinemia is rare and is due to an abnormal hemoglobin structure or a deficiency in a reducing enzyme. These mutations can be autosomal recessive or dominant and include disease entities such as cytochrome b5 reductase deficiency and hemoglobin M disease.^2^ Acquired cases of methemoglobinemia are more prevalent, although the incidence is not well defined. Typically, medications and other chemical substances with oxidative potential are the cause. Common medications with oxidative potential include local anesthetics (lidocaine, benzocaine, and prilocaine), sulfa-containing medications, and dapsone.^3^ Infants are particularly susceptible to methemoglobinemia when facing an oxidative stress due to the immaturity of their enzymatic systems and increased potential for accidental overdose.^3^

Methemoglobinemia presents with hypoxia causing cyanosis, which can be a diagnostic challenge in a neonate. The possible causes of hypoxia in a neonate are extensive and include, but are not limited to, cyanotic congenital heart disease, sepsis, pneumonia, congenital airway or pulmonary anatomic abnormalities, pulmonary interstitial abnormalities, neurologic conditions, toxidromes, and hemoglobinopathies. While pulmonary etiologies are the most common cause of hypoxia and central cyanosis in an otherwise healthy child, methemoglobinemia should be considered in patients with exposure to oxidizing agents or a family history of congenital methemoglobinemia.

Infants experiencing acute methemoglobinemia may develop a physical exam finding of central cyanosis, hypoxia, increased work of breathing, altered mental status, respiratory depression, and seizures.^3^ The end result may be death.^3^ Central cyanosis appears differently depending on the pigmentation of a child's skin. Children with more skin pigmentation have bluish-grey color changes most noticeable on the tongue and mucosal membranes. Children with less skin pigmentation have blue color changes most noticeable on the tongue, mucosal membranes, and perioral area. A key indication of possible methemoglobinemia is a peripheral oxygen saturation detected by pulse oximetry that does not improve with oxygen administration. Upon phlebotomy, the blood remains dark brown in color after exposure to oxygen.^3^ The arterial blood gas is typically within normal limits. A peripheral capillary oxygen saturation (SpO2) monitor often underestimates hypoxia in the setting of high levels of methemoglobin, so this should not be relied upon for clinical monitoring.^4^ CO-oximetry should be used to detect the percentage of methemoglobin. It does this by measuring specific wavelengths of light corresponding to methemoglobin (660 nm and 940 nm).^4^

A majority of cases of methemoglobinemia resolve with supportive care only. More severe cases, however, can be treated with methylene blue to reduce the ferric ions of the heme molecules back to their ferrous state. This allows for appropriate oxygen dissociation from hemoglobin.^5^ The most commonly accepted methemoglobin level requiring treatment is a level greater than 20% of hemoglobin.^6^ If the patient is significantly symptomatic, however, methylene blue may be administered with lower levels. It is typically dosed at one to two milligrams per kilogram.^6^ The overall efficacy of methylene blue in the treatment of methemoglobinemia varies. The primary contraindication to methylene blue use is glucose-6-phophate dehydrogenase deficiency due to the risk of hemolysis.^6^

This simulation case presents a rare but potentially lethal toxic exposure of topical lidocaine, which induces methemoglobinemia. Content experts in pediatric emergency medicine (PEM) and toxicology created this simulation and originally designed it for PEM fellow education. It is also relevant to other health professionals who care for acutely ill children, including pediatric residents, emergency medicine residents, family medicine residents, medical students, advanced practice providers, and nurses. Learners must evaluate and stabilize an infant presenting with central cyanosis and respiratory distress of unknown etiology. This simulation serves as an educational tool to accommodate this need. Importantly, toxidromes due to medication are a content domain for the PEM certification examination.^7^

This scenario encourages active learning and integration of previously acquired knowledge, skills, and behaviors as learners must evaluate and stabilize an infant presenting with central cyanosis and respiratory distress of unknown etiology. While the underlying etiology is lidocaine-induced methemoglobinemia, participants must work as a team to use a systematic approach to care for an acutely ill pediatric patient, a theme common to the emergent care of children. Through this scenario, learners must evaluate a cyanotic and hypoxic infant, implement initial resuscitation interventions, develop a broad differential diagnosis, and implement a diagnostic plan leading them to the eventual diagnosis of methemoglobinemia. While other simulation cases addressing methemoglobinemia exist, this scenario is unique in its focus on a neonate necessitating a broad differential diagnosis.^8,9^ This simulation-based curriculum can be used independently or in series with other sessions from the Pediatric Toxidrome Simulation Curriculum.^10–15^

## Methods

### Development

We created this simulation to teach learners a systemic approach to PEM care and promote interpersonal dialogue and communication. The primary goals of the case included working as a team evaluating a neonatal patient with hypoxia and central cyanosis, implementing initial steps of stabilization, thinking through a differential diagnosis, and identifying signs and symptoms of methemoglobinemia.

This simulation scenario was created by PEM attendings with expertise in simulation and curriculum design. A toxicologist provided additional support in the case creation as a content expert. This case was initially created for PEM fellows as part of their recurring simulation-based education. It was also implemented with other members of the emergency department (ED) clinical team who provide medical care to pediatric patients in an ED setting. No specific preparation was required by the learners prior to participation in the simulation. Participants had to have prerequisite knowledge about rapid evaluation and management of neonatal respiratory distress and skills in airway management, which were expected skills for medical personnel working in a pediatric ED. The instructors were provided with the simulation scenario (Appendix A), simulation environment preparation (Appendix B), diagnostic images including electrocardiogram and chest radiograph (Appendix C), teamwork and communication glossary (Appendix D), debriefing guide (Appendix E), and participant evaluation form (Appendix F). Educational slides (Appendix G) were reviewed with participants after the simulation to augment learning and reinforce important concepts. Assessment of the curriculum focused on levels 1 and 2 of Kirkpatrick's model.^16^

### Equipment/Environment

All sites conducted this simulation in a pediatric ED or simulation laboratory with a high-fidelity, infant-sized manikin. The case could also be run in other clinical locations and/or using a low-fidelity manikin. Specific environmental preparation was available in Appendix B. We began the simulation with the manikin on the hospital bed after being brought to the ED by parents. Participants were told that he was in respiratory distress. There was no intravenous access. Equipment and medications commonly encountered in the ED environment were available as outlined in Appendix B. Printouts of an electrocardiogram and chest radiograph were available upon request, all of which showed normal findings (Appendix C).

If using a low-fidelity manikin, facilitators can report vital signs verbally or write them in an area easily seen by all participants. Physical exam findings can be verbally described during the evaluation. The central characteristic of this simulation, cyanosis, can be verbally communicated to the participants. Additionally, moulage can be considered to make the patient appear to be cyanotic.

### Personnel

This simulation scenario was performed with seven to 16 learners during each session. Participants were oriented to the simulation, including the manikin and safe learning environment principles. Available roles included three physician/medical provider roles, four nursing roles, and two instructor roles. For sessions in which interdisciplinary team members were not present, physicians filled these roles. The optimal number of participants was seven (three physician/medical provider roles and four nursing roles). The optimal number of instructors was two. Roles for pharmacists and respiratory therapists could be incorporated into the simulation if personnel were available. If the scenario was completed with an interdisciplinary team, personnel maintained the roles of their employment; for example, a fellow would be in the role of a physician, and a nurse would be in the role of a nurse. If the participant group was smaller than the typical clinical team, the simulation would be completed with a minimum of three participants fulfilling physician roles and one facilitator fulfilling an instructor role. This would, however, decrease the opportunity to practice teamwork and communication skills. For larger groups, such as the group of sixteen participants, learners who did not have a designated role were recommended to observe and participate in the didactics and debrief. Instructors were all PEM-trained physicians with experience in medical simulation.

### Implementation

This scenario was implemented with a total of 56 participants (including 18 PEM fellows) at four sites across the United States. Each site performed the scenario between one and three times with different groups of learners. The scenario was described in Appendix A. The scenario started with the infant in an ED exam room after having been rushed there from triage. The ED team was called to bedside to evaluate the child with central cyanosis and respiratory distress. The team member assigned to the role of nurse applied the monitors, which showed an oxygen saturation of 85% via the simulation monitor. This alerted the team to make attempts to support the child's breathing during the primary survey. A facilitator or embedded participant played the role of the parent and, upon request, gave the team history, which helped narrow the differential diagnosis to methemoglobinemia induced by lidocaine exposure. Labs as requested in addition to use of CO-oximetry confirmed the diagnosis and appropriate treatment. At participant request, electrocardiogram and chest radiograph (Appendix C) were available and unremarkable. Appropriate sign-out to the neonatal intensive care unit was expected at the conclusion of the scenario. Facilitators were provided with teamwork and communication tools prior to each session, found in Appendix D. This allowed for standardized terminology to debrief the scenario in order to optimize the educational experience. The debriefing guide was provided in Appendix E to allow facilitators to provide effective feedback and participants to obtain optimal learning from the experience. A slide-based didactic to aid in the debriefing was provided in Appendix G. An evaluation form for participants to complete after case participation was provided in Appendix F and could be used to adjust the case in the future.

### Assessment

The scenario was facilitated and debriefed by PEM physicians experienced in simulation. The participants were PEM fellows, emergency medicine residents, pediatric and emergency medicine interns, PEM RNs, PEM attendings, a respiratory therapist, and a physician assistant. Facilitators provided pediatric resuscitation and content expertise as it related to managing a cyanotic infant and methemoglobinemia. Facilitators also provided participant performance feedback in accordance with the learning objectives. Following the scenario debriefing, participants completed the evaluation form (Appendix F) to give facilitators feedback on the relevance, realism, and overall learning experience of the simulation scenario. The evaluation form included statements rated on a 5-point Likert scale (1 = *strongly disagree,* 2 = *disagree,* 3 = *neutral,* 4 = *agree,* 5 = *strongly agree*) to assess the success of the simulation in addressing the learning objectives and to report confidence managing a similar scenario after participation. Respondents had the opportunity to provide additional feedback on the clinical impact and ideas for scenario improvement through free text and open-ended questions, including “Can you list/describe one or more ways this simulation session will change how you do your job?”, “How can we improve this scenario?”, and “Additional comments.” Median Likert scores were calculated for each item on the survey.

### Debriefing

We used the tools in Appendices D and E to assist the facilitation debrief sessions after the simulations. These guides provided an outline for leading a debriefing session. We recommend that these sessions begin by encouraging participants to share their overall reflection of the scenario, followed by a structured discussion outlined in Appendix E. The discussion included topics of teamwork and communication (Appendix D) as well as reiteration of diagnostic and management skills.

## Results

The scenario was facilitated by experienced PEM simulation faculty across four institutions who provided content expertise and constructive feedback to learners with regard to the learning objectives. A total of 56 individuals participated in the simulations and completed the postsimulation survey. Participants included PEM fellows, emergency medicine residents, pediatric and emergency medicine interns, a physician assistant, a respiratory therapist, PEM nurses, and PEM attendings. Numbers of participants from each discipline and level of training are described in Table 1. The largest portion was PEM fellows, who made up 32% of participants. Emergency medicine residents composed the second largest group of 25% of the total participants. The smallest simulation was completed with seven participants, and the largest was completed with 16 participants. In groups with more participants than available roles, learners without assigned roles observed the simulation and participated in the didactic and debrief. Table 2 summarizes participants' experience related to the simulation case and session facilitation. Overall, participants strongly agreed that the simulation was relevant to their work, realistic, and effective in teaching the objectives and that it promoted team reflection. Table 3 summarizes participant self-appraisal after participation related to the learning objectives. Overall, participants were confident in their ability to perform the designated learning objectives on subsequent patients.

**Table 1.**
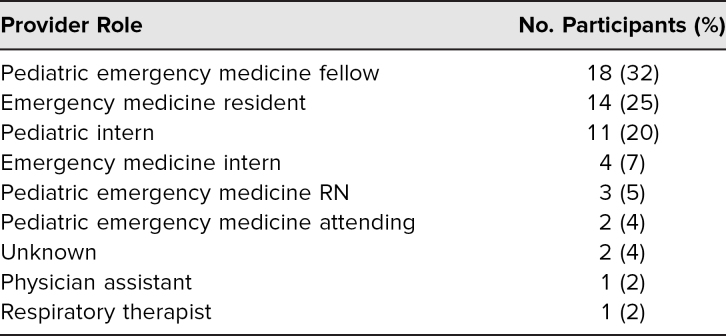
Simulation Participants (*N* = 56)

**Table 2.**
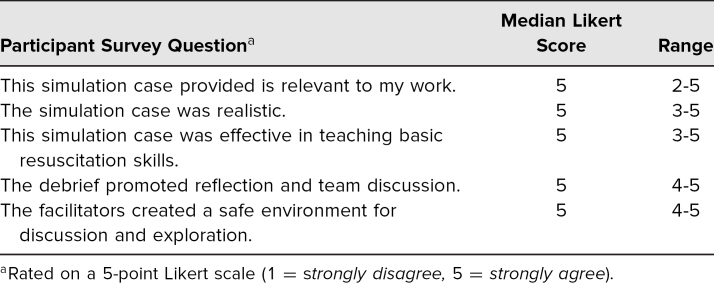
Participant Experience During the Simulation Session (*N* = 56)

**Table 3.**
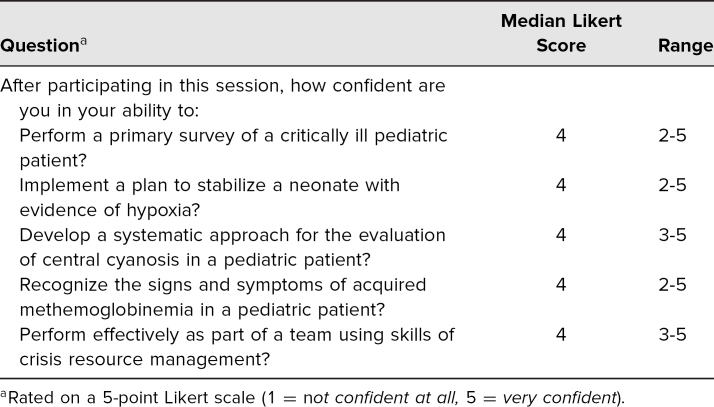
Participant Clinical Confidence After Participating in the Session (*N* = 56)

Each participant was given the opportunity to respond to the following question: “Can you list/describe one or more ways this session will change how you do your job?” Responses primarily discussed improved differential diagnosis building with respect to a hypoxic and cyanotic pediatric patient and further understanding of methemoglobinemia pathophysiology. Responses included the following:

• “Critically think about cyanosis in a patient with a normal venous blood gas.”
• “Recognize that seemingly benign medications (to parents) can have true complications.”
• “Thinking of methemoglobinemia as part of my differential diagnosis for cyanosis.”
• “Understanding of indications for methylene blue treatment.”
• “Encourage me and widen differential diagnosis and gather more extensive history.”
• “Knowing availability of CO-oximetry within my practice.”

Of note, one PEM fellow, who had participated in the first iteration of this case, encountered a child with cyanosis and desaturations unresponsive to supplemental oxygenation several months after completing the simulation. He attributed his quick consideration of and evaluation for methemoglobinemia to having participated in this simulation session.

Participants' suggestions for improvement to the session included making the SpO2 reading 85%-88%, keeping learner groups to fewer than 10 participants, and allowing more time for the session and debrief.

## Discussion

Due to widespread availability of topical anesthetics and the severity of the potential complication of methemoglobinemia, health care providers caring for children in ED settings must be able to recognize and treat this rare but fatal complication. This simulation provides an opportunity for PEM fellows to review the differential diagnosis of a hypoxic and cyanotic infant; identify history, symptoms, and physical exam findings consistent with lidocaine toxicity and resultant methemoglobinemia; and strengthen team communication skills. While other curricula related to lidocaine toxicity have appeared in *MedEdPORTAL,* none are specific to the evaluation of a hypoxic and cyanotic infant.^8,9^ This curriculum provides a modality for learners to expand their differential diagnosis to include lidocaine toxicity in hypoxic infants. For many pediatric and emergency medicine providers, this scenario is rare but life-threatening. Prompt recognition and appropriate management are important. Additionally, the scenario allows learners to enhance teamwork and communication skills in a controlled environment while also providing a dedicated space to debrief team dynamics.

Multiple learners have participated in the simulation, which is necessary to refine and adapt the material. The simulation case was adjusted after the initial iteration to include an oxygenation saturation typical of methemoglobinemia. Larger groups of learners identified a benefit to completing the simulation in smaller groups. While that was not always possible due to time constraints and predetermined learner group size, subsequent sessions should be held with groups small enough to allow ample opportunity for participation and engagement. If group sizes must be larger than the number of personnel required to run the case, some participants should be assigned an observer role with a special focus on providing observations and feedback to the other participants. This allows the simulation to maintain the realism of a resuscitation. There should be sufficient participants and facilitators to ensure the crucial roles are filled.

A primary limitation of this activity is the difficulty of incorporating all providers typically present on a resuscitation team due to scheduling constraints of nurses, respiratory therapists, and pharmacists. While the simulation was created for PEM fellows, the interdisciplinary teamwork and debrief are enhanced by the incorporation of health care providers from various disciplines. In the future, this simulation should prioritize PEM fellow involvement with incorporation of disciplines who typically care for children in the ED. While PEM fellows make up the largest group of participants for this simulation, they constitute only 32% of participants. This is due to availability of PEM fellows at the institutions included in the evaluation of the simulation and the simultaneous desire to include all health care professionals working in the ED. A limitation of the evaluation process is the reliance of the evaluations on participant perception of improvement of skills after completing the session. Teach-back methods and testing/retesting knowledge, behaviors, and skills through additional simulations have not been evaluated. The immediate feedback provided by learners, however, has been reviewed and assessed for integration in future iterations, as described in preceding paragraphs. Additionally, to better define the time frame within which these knowledge and skills are obtained and reevaluated, future iterations of this simulation should include a time frame associated with each objective.

## Appendices

Simulation Case.docxEnvironment Preparation.docxImages.pptxTeamwork and Communication Glossary.docxDebriefing Guide.docxEvaluation Form.docxDidactics.pptx
All appendices are peer reviewed as integral parts of the Original Publication.
