# Supplementary material for: Pediatric Toxidrome Simulation Curriculum: Lidocaine-Induced Methemoglobinemia
Source: MedEdPORTAL. 2021 Jan 28;17:11089. doi: 10.15766/mep_2374-8265.11089 (PMC7842087; doi:10.15766/mep_2374-8265.11089)
Supplement: Supplementary file 1 — Simulation Case.docxEnvironment Preparation.docxImages.pptxTeamwork and Communication Glossary.docxDebriefing Guide.docxEvaluation Form.docxDidactics.pptx [file mep_2374-8265.11089-s001.zip › A. Simulation Case.docx]

| Appendix A: Lidocaine Induced Methemoglobinemia Simulation Case  SIMULATION CASE TITLE: Pediatric Emergency Medicine Simulation Curriculum: Lidocaine Induced Methemoglobinemia  AUTHORS: Chelsea Del Rosso, MD, Anita Thomas, MD, MPH, Nicole Hardy, MD, Scott Connelly, MD, Ulysses Davila, MD, Jean Pearce, MD, Suzan Mazor, MD, Rebekah Burns, MD | |
| --- | --- |
| PATIENT NAME: Kevin  PATIENT AGE: 4 weeks old  PATIENT WEIGHT: 4 kg  CHIEF COMPLAINT: “My baby’s lips are changing color!” | |
|  | |
| Brief narrative description of case | Kevin is a 4-week-old previously healthy boy who has acute onset of jerking movements, increased work of breathing and color change of his lips. His parents bring him into the emergency department (ED) for evaluation. Initially, the patient has increased work of breathing and central cyanosis notable on his oral mucous membranes with low peripheral capillary oxygen saturation. He is otherwise hemodynamically stable and appears fatigued. A thorough evaluation reveals that the patient has methemoglobinemia secondary to lidocaine use after his circumcision.  Anticipated interventions include primary and secondary surveys, evaluation of hypoxia in a child, including supporting airway, breathing, circulation, establishing vascular access, obtaining appropriate laboratory values, and imaging. Learners may consider consultation with neurology, toxicology, and neonatology. |
| Primary Learning Objectives | By the end of this module, the learner will be able to:   - Perform a primary survey of a critically ill pediatric patient - Implement a plan to stabilize a hypoxic and cyanotic neonate - Develop a systematic approach for the evaluation of hypoxia and central cyanosis in a pediatric patient - Describe the signs and symptoms of acquired methemoglobinemia in a pediatric patient - Manage a pediatric patient with acquired methemoglobinemia - Demonstrate teamwork and communication skills in a resuscitation setting |
| Critical Actions | - Perform initial primary survey (ABCDE: airway, breathing, circulation, disability, exposure) - Perform initial evaluation and stabilization of a patient with cyanosis (signifying hypoxia) and respiratory distress   - Support oxygenation and ventilation   - Obtain intravenous or intraosseous (IV/IO) access   - Obtain and interpret relevant studies - Perform secondary survey and recognize potential lidocaine exposure as part of SAMPLE history - Perform as part of an effective team   - Assign roles to accomplish multiple tasks simultaneously   - Communicate clearly with team members   - Discuss likely diagnosis and appropriate management amongst participants - Communicate openly with the parent even if there is uncertainty |
| Learner Preparation | Suggested references may be provided to learners before or after participation depending on needs and specific local goals   - Introduction to the concept of specific team roles in resuscitation   - Farah MM, Tay K-Y, Lavelle J. A general approach to ill and injured children. In: Shaw KN, Bachur RG, eds. Fleisher & Ludwig’s Textbook of Pediatric Emergency Medicine. 7th ed. Philadelphia, PA: Wolters Kluwer; 2016:1-8. - General knowledge of PALS, APLS, ACLS, or other general resuscitation protocols - Introduction to TeamSTEPPS   - Appendix D   - TeamSTEPPS: national implementation. Agency for Healthcare Research and Quality Website. http://teamstepps.ahrq.gov. Accessed September 3, 2019. - General knowledge about acquired methemoglobinemia and approach to a child with central cyanosis   - Fleisher & Ludwig Section II: Signs & Symptoms. Chapter 16: Cyanosis (7th edition).   - Skold A, Cosco DL, Klein R. Methemoglobinemia: pathogenesis, diagnosis, and management. *South Med J*. 2011;104(11):757-761. doi:10.1097/SMJ.0b013e318232139f   - Steinhorn, R. Evaluation and management of a cyanotic neonate. Clin Pediatr Emerg Med. 2008 Sep; 9(3): 169–175. doi: [10.1016/j.cpem.2008.06.006](https://dx.doi.org/10.1016%2Fj.cpem.2008.06.006) |

| Initial Presentation | | | |
| --- | --- | --- | --- |
| Initial vital signs | *Heart rate* 163 beats per minute  *Blood pressure* 72/45  *Oxygen saturation* 85% on room air  *Respiratory rate* 60 breaths per minute  *Temperature* 36.9 ^o^C | | |
| Overall Appearance | Kevin is in the pediatric emergency department and is being held by his parents. He is in a standard emergency department room. He is tachypneic, fatigued, and has a weak cry. He is not on monitors. | | |
| Actors and roles in the room at case start | This scenario requires a minimum of 3 participants to fill the physician/medical provider roles and one facilitator to fill the instructor #1 role. Additional roles can be filled by additional participants or by facilitators. Participant roles may be adjusted based upon the typical team structure of resuscitation teams within the institution.  Doctor #1: Team Leader physician  Doctor #2: Airway physician  Doctor #3: Survey physician  Nurse #1: Medication Administration Nurse (RN)  Nurse #2: Medication Preparation RN (optional)  Nurse #3: Documenting RN (optional)  Nurse #4: Circulating RN (optional)  Instructor #1: Simulation facilitator who will lead the debrief and can act in the role of parent if personnel is limited  Instructor #2: If a 2^nd^ instructor is available, cast them as “parent,” available to answer questions and assist with debrief. If a 2^nd^ instructor is not available, the Instructor #1 can play the role of parent as well. (optional) | | |
| HPI | Information volunteered by the parent: “We were on the way home from the doctor’s office “We were riding in the car with him when he started jerking his arms and his legs. His lips were changing color. I got in the back seat with him and tried to wake him up and he started to breath faster so we brought him straight here.”  When asked about events leading up to the event (SAMPLE):  Signs/symptoms (sx)- Kevin is a 4-week-old male presenting with the chief complaint of “lips changing color”. His symptoms started 15 minutes prior to presentation in the emergency department. “We were riding in the car with him when he started jerking his arms and his legs. His lips looked like they were changing color - turning dark blue. I got in the back seat with him and tried to wake him up and he started to breathe faster so we brought him here.” He has not had any fevers, congestion, cough, emesis, diarrhea or rash leading up to this. He had been in his normal state of health.  Allergies- none  Medications- none  Past Medical History: He was born full term without any pregnancy or birth complications. He has no previous hospitalizations. His immunizations are up to date.  Last meal: “He had a bottle of formula 2 hours ago.” If asked, the formula was mixed appropriately.  Events preceding: He had no symptoms prior to the event and was behaving normally. “He had a circumcision yesterday.” There has not been excessive bleeding since the procedure.  If asked for medications given during circumcision: “He was given a medication for pain, but I don’t know what it was called. It was put directly on top of his penis.” If asked if they are continuing to put anything on the site, "We did take the little tube of the medicine. It seemed to help the pain. I put it on before bed last night and once this morning."  If asked for review of systems:  Positive for: Shortness of breath and fatigue  Negative for: Fever, rhinorrhea, nasal congestion, injected conjunctiva, difficulty feeding, vomiting, diarrhea, and decreased urine output.  If asked for social history:  He lives at home with us (mother and father). No one is sick at home and he has been home with us since he was born. He is only formula fed. There are no pets in the home, and he doesn’t have any older siblings. He is not in daycare and stays home with his father.  If asked for family history:  “I (mother) have type II diabetes and father is healthy.” No history of seizures or congenital heart disease if specifically asked. | | |
| Past Medical/Surgical History | Medications | Allergies | Family History |
| Medical: None  Surgical: Circumcision | Topical medication during circumcision. No regular medications. | None | Mother has type II diabetes. |
| Physical Examination (primary survey) | | | |
| General | Alert, crying | | |
| HEENT | Patent airway, Head is normocephalic and atraumatic without bruising or step off. No nasal discharge and oropharynx is clear. Dark blue-gray discoloration of lips and tongue. | | |
| Neck | Full range of motion. | | |
| Lungs | Clear to auscultation bilaterally. No wheezes or rhonchi. Moderate subcostal retractions, tracheal tug, nasal flaring. | | |
| Cardiovascular | Normal S1 and S2, no murmur, no gallop, 2+ femoral pulses, no brachial-femoral delay, capillary refill (CR) <2 seconds, warm skin. | | |
| Abdomen/GI | Abdomen soft, non-tender, without masses; no guarding. | | |
| Neurological | GCS is 14 (E4, M6, V4). Pupils are equal and reactive to light bilaterally. Normal and symmetric muscle bulk, tone. No abnormal twitching or tonic-clonic movements. | | |
| Skin | No rash. No acral cyanosis. No bruising or petechiae. | | |
| GU | Gauze in place over penis without active bleeding. | | |

| Instructor Notes - Changes and CASE Branch Points | | |
| --- | --- | --- |
| Intervention / Time point | Change in Case | Additional Information |
| Patient is placed on monitors or vitals are obtained | Heart rate (HR) 163 beats per minute  Oxygen saturation (SpO2) 80%  Blood Pressure (BP) 72/45 mmHg  Respiratory Rate (RR) 60 breaths per minute  Temperature (T) 36.9 degrees Celsius | Patient is sleepy but arousable. There are no jerking movements at this time. |
| Intravenous (IV) line placement | If requested, obtained on first attempt |  |
| Blood glucose level is requested. |  | Blood glucose=80 |
| Capnography end-tidal CO2(ETCO2) monitor placed if available. | Waveform with CO2 at 38 mmHg, other vital signs (VS) are unchanged. |  |
| Supplemental oxygen provided by any method (including BMV) at any flow. | SpO2 remains 85%. | The team should reposition the airway, and/or place nasopharyngeal airway. There is no change in the SpO2 or work of breathing. |
| After the primary survey, the parent states: “Why is my child’s color different?!” |  | *Team member to explain what is occurring (interventions, patient status) in layman's terms, e.g. “We are trying to help figure out why his color is changing and working hard to breath. We are giving him oxygen because his oxygen level is low.”*  *If not already asked, this is a good place to obtain further history from the parent (see HPI).* |
| If the team does not discuss a differential diagnosis after completion of primary and secondary surveys the parent askes, " What do you think is going on?” |  | *Leader may elicit input from the team to expand differential diagnosis.*  Recognize finding of central cyanosis and hypoxia consider a differential diagnosis:  Upper airway obstruction:  -Foreign body aspiration, trauma, bacterial tracheitis, epiglottitis  Lower airway/parenchymal/interstitial disease:  -Bronchopulmonary dysplasia, bronchiolitis, pneumonia, pneumothorax  Pulmonary vasculature disorder:  -Pulmonary hypertension  Congenital heart disease:  -Cyanotic heart lesions and  ductal dependent lesions such as coarctation of the aorta  -Ventricular septal defect causing heart failure  Infectious disease:  -Primary infection (pneumonia, meningitis, urinary tract infection)  Neurologic:  -Seizure: primary seizure disorder or central nervous system anomaly  Toxicology:  -Toxin ingestion  -Toxin exposure (i.e. inhalation or injection) causing methemoglobinemia |
| Participant requests point-of-care (POC) testing, serum labs, and/or urine labs: glucose, blood gas, electrolytes (sodium, potassium, chloride, ionized calcium (iCal), bicarbonate), blood urea nitrogen (BUN), creatinine, hemoglobin, hematocrit, complete blood count (CBC), blood culture, urinalysis with culture, urine toxicology.  Consider ordering lorazepam to have at bedside due to twitching noted in the car and concern for seizure. |  | POC labs announced 1 minute later:  Glucose: 115 mg/dL  CBG: 7.32/49/75/-1  Electrolytes: 138/4.5/104/25  iCal: 1.13 mmol/L  BUN: 5 mg/dL  Creatinine: 0.2 mg/dL  Hemoglobin: 10.5 g/dL  Hematocrit: 31%  Serum labs announced 3 minutes later.  CBC: 8.2>10.5/31<250  If an arterial blood gas is requested, the team will be unable to obtain arterial access. CBG is the only blood gas that can be obtained.  If urinalysis or urine toxicology is requested, bedside RN should be instructed to obtain a catheterized urine specimen due to concern for infection. Urinalysis and toxicology results will not return during initial resuscitation. |
| Lidocaine level and methemoglobin level to be ordered if requested. Methemoglobin results in 15 minutes (during sign out of patient at conclusion of case). |  | Labs sent and pending, |
| Electrocardiogram (ECG) requested. | ECG: normal sinus rhythm | See Appendix C. |
| If requesting chest and/or abdominal radiograph |  | Nurse replies, “X-ray is in route.” |
| Head computed tomography (CT) requested. |  | Nurse replies, “There is another patient in head CT right now, it will be at least 5 minutes until they are available. The patient does not appear stable enough to go to the scanner.” |
| Echocardiogram requested. |  | Nurse replies, “Cardiology in route to perform an echocardiogram. It will be at least 15 minutes.” |
| Electroencephalogram (EEG) requested. |  | Nurse replies, "EEG is unavailable right now." |
| 5-7 MINUTES INTO THE CASE |  |  |
| Reassess airway, breathing, circulation (ABCs). | Unchanged, no improvement in retractions or hypoxia even if oxygen is being delivered. | If CO-oximetry or methemoglobin level has not been ordered, a nurse should state, "Why isn't his oxygen level improving?" |
| Team requests antibiotics for concern of sepsis. | Antibiotics given without incident. | Lumbar puncture should be deferred until stability is achieved. |
| Facilitator reports that x-ray has arrived and provides the team with an image of chest x-ray and/or abdominal. | Team provided an image of a normal chest and abdominal x-ray (if requested). | See Appendix C. |
| 7-10 MINUTES INTO THE CASE |  |  |
| Repeat assessment. | Unchanged*.* | If CO-oximetry or methemoglobin level has not been ordered, a nurse should state, "Why isn't his oxygen level improving?" |
| Apply CO-oximetry, if requested. |  | CO-oximetry reading if requested shows 20% methemoglobin and 80% oxyhemoglobin. |
| If the team intubates the patient | No changes in oxygen saturation. | Nurse should state, "Why isn't his oxygen level improving?" |
| Team consults NICU, PICU or pediatric ED for continued monitoring and intervention. | Oxygen saturation increases to 83% | Begin sign out to the accepting team. During the sign out, Instructor #1 interrupts with methemoglobin lab result if requested earlier in the case. The methemoglobin fraction is 20% confirming the diagnosis of methemoglobinemia due to lidocaine.  If no NICU, PICU or pediatric ED consult is made, Instructor #1 prompts the team leader for a summary and disposition. |
| If toxicologist is consulted regarding methemoglobinemia |  | Consult recommends continued supportive care given slight improvement in oxygen saturation.  Methemoglobin level is 20% of total hemoglobin with symptoms of respiratory distress and cyanosis. If the patient had continued severe symptoms due to methemoglobin, recommend treating with methylene blue at 1-2mg/kg over 5 minutes due to no history of G6PD deficiency. Due to the patient's slow resolution of symptoms, a decision can be made to not administer methylene blue and this would be appropriate as well. |

Ideal Scenario Flow

The learners enter the room to find a child in a hospital gown with physical exam findings of central cyanosis and in respiratory distress. They immediately assign team roles. Monitors are placed on the patient. They assess the patient’s overall appearance and bedside monitor readings. They determine the patient is cyanotic, in respiratory distress, and hypoxic. The team considers the differential diagnosis of central cyanosis and hypoxia in an infant patient. Supplemental oxygen is placed on the patient, vascular access is secured, and initial labs are obtained. These initial labs are unremarkable, and the SpO2 does not change with supplemental oxygen. The team recognizes the need to evaluate a broad differential including but not limited to upper airway obstruction (foreign body aspiration, trauma, bacterial tracheitis, epiglottitis), lung disease (bronchopulmonary dysplasia, bronchiolitis, pneumonia), neurologic disease (seizures, intracranial bleed), pulmonary vascular disorder (pulmonary hypertension, pulmonary hemorrhage), hematologic pathology (methemoglobinemia), cyanotic cardiac lesion or other congenital heart disease progressing to heart failure (aortic coarctation, hypoplastic left heart, atrioventricular canal defect), ingestion, injuries due to non-accidental trauma, and infection causing septic shock. They obtain blood cultures, complete blood count, glucose, blood gas, electrolytes, and, based on history, and a methemoglobin level. They obtain electrocardiogram, chest x-ray, possibly an abdominal x-ray, and discuss the need for echocardiogram, head CT without contrast, and lumbar puncture. A lumbar puncture and head CT should be delayed until stability is achieved. EEG may be considered but will not be able to be performed during the initial resuscitation.

The patient is reassessed multiple times (at least three) during the scenario and ideally after each intervention. Chest x-ray and abdominal x-rays are unremarkable, and ECG is normal sinus rhythm is within normal limits. Echocardiogram shows normal cardiac function and structure. Glucose, electrolytes, blood gas, and CBC are within normal limits. EEG, CT, and lumbar puncture are unable to be performed during time of case. Lidocaine level and methemoglobin level are elevated, but these do not return during resuscitation and can be revealed after completion of simulation. Expert consultation such as NICU, PICU or pediatric emergency medicine specialist to assist with disposition is recommended. Immediately after the learner provides a specialist with a case summary and consult question, the methemoglobin level returns elevated. This prompts a team discussion about how to manage the patient next. Methylene blue is considered and the need for the medication is discussed. The team should admit the patient to the hospital for further observation and resolution of all symptoms.

Anticipated Management Mistakes

1. Failure to recognize central cyanosis and formulate a differential diagnosis for central cyanosis and hypoxia in an infant.
   1. We found that some learners had trouble generating a differential diagnosis that included pathology beyond pulmonary or cardiac etiologies. If learners were demonstrating premature diagnostic closure, the facilitator should ask “Is there anything else that could be going on?” to elicit the creation of a broad differential.
2. Failure to recognize toxin exposure in an infant causing cyanosis and hypoxia.
   1. Some learners did not take an adequate history to recognize lidocaine toxicity. If the caregiver was not asked about medications, the parent could prompt “Do you think this is because of the circumcision?” They could further prompt, “Did they give him something that caused this?”
3. Failure to recognize no improvement in respiratory status after application of oxygen and high flow nasal cannula, which can assist in guiding the diagnosis.
4. Failure to recognize altered mental status of an infant.
5. Dosing error and failure to adhere to dosing references.
   1. A Broselow tape and PALS cards can be available for learners to utilize as references.
