# Supplementary material for: Pediatric Toxidrome Simulation Curriculum: Lidocaine-Induced Methemoglobinemia
Source: MedEdPORTAL. 2021 Jan 28;17:11089. doi: 10.15766/mep_2374-8265.11089 (PMC7842087; doi:10.15766/mep_2374-8265.11089)
Supplement: Supplementary file 1 — Simulation Case.docxEnvironment Preparation.docxImages.pptxTeamwork and Communication Glossary.docxDebriefing Guide.docxEvaluation Form.docxDidactics.pptx [file mep_2374-8265.11089-s001.zip › B. Environment Preparation.docx]

**Appendix B: Lidocaine Simulation Environment Preparation**

Before each simulation, ensure the anticipated resuscitation equipment is available for the team’s use. The medications and equipment available should reflect what is actually available to participants in real practice. Not all medications or equipment are necessary for the simulation, as anticipated with ideal flow, but reflect what is often found in a pediatric emergency medicine department for the care of critically ill patients.

**Resources**

Pediatric resuscitation and medication references (e.g. PALS, reference cards)

Patient Weight Estimator such as a Broselow tape

Documentation forms

**Personal Protective Equipment**

Staff gowns

Gloves

Mask and face shields

**Medications (consider having all or only a limited number of medications available)**

Adenosine

Amiodarone

Bicarb

Calcium Chloride or Calcium gluconate

Epinephrine 1:10,000

Epinephrine 1:1,000

Etomidate

Lipid emulsion

Ketamine

Lidocaine

Lorazepam

Magnesium

Methylene Blue*

Midazolam

Normal Saline/Lactated Ringers

Potassium Chloride

Procainamide

Rocuronium

Sodium bicarbonate

Succinylcholine

**Equipment (consider having some or all equipment based on usual clinical environment)**

Bag-valve-mask system, multiple size masks

Bedside blood sample processors: glucose, electrolytes, gases*

Bedside ultrasound machine

CO-oximetry*

Code cart

CPR stool and backboard

Defibrillator / AED

End-tidal CO2 colorimeter

Endotracheal tubes- 3.0, 3.5, 4.0, 4.5, 5.0, 5.5, 6.0, 6.5, 7.0, 7.5 cuffed or uncuffed, stylets

High flow nasal cannula system*

Intraosseous needles, 2 sizes

IV/Angiocath, various sizes*

IV pumps, pressure bags/ blood product pumps

IV tubing/blood product tubing and filters

Laryngoscope, Miller and Mac blades, multiple sizes

Monitors: blood pressure cuff, heart rate monitor leads, oximeter probe, defibrillator cables*

Nasal and oral airways, multiple sizes

Nasogastric tube(s)

Oxygen delivery device (nasal cannula, simple mask, or non-rebreather mask) *

Oxygen source – on wall or cylinder*

Shoulder roll*

Simulator manikin in hospital gown or clothing, on bed with patient identification band*

Specimen tubes*

Stethoscopes*

Suction device

Syringes, multiple sizes

Thermometer, temperature probe*

*Medications and equipment most likely to be used during simulation.
