# Supplementary material for: Pediatric Toxidrome Simulation Curriculum: Lidocaine-Induced Methemoglobinemia
Source: MedEdPORTAL. 2021 Jan 28;17:11089. doi: 10.15766/mep_2374-8265.11089 (PMC7842087; doi:10.15766/mep_2374-8265.11089)
Supplement: Supplementary file 1 — Simulation Case.docxEnvironment Preparation.docxImages.pptxTeamwork and Communication Glossary.docxDebriefing Guide.docxEvaluation Form.docxDidactics.pptx [file mep_2374-8265.11089-s001.zip › E. Debriefing Guide.docx]

**Appendix E: Lidocaine-Induced Methemoglobinemia Simulation Debriefing Materials**

**Debriefing Overview**

*We believe that reflective learning occurs in the DEBRIEF. It’s an opportunity for learners to reflect on their medical decision making, technical, teamwork and communication skills. The ultimate goal is to identify gaps and potential solutions to close those gaps, leading to improved patient safety and quality of care.*

**Framework for debriefing:**

We model our debriefing after PEARLS ^1^. Each debrief typically has 4 phases:

- - - **Reactions phase**- opportunity for learners to express their emotional experience, where they may reveal key areas that are important to them
    - **Description phase-** opportunity for learners to summarize key events in the scenario to ensure that educators and learners are on the same page
    - **Analysis phase-** opportunity to explore the medical decisions, technical, teamwork and communication performance of the team
    - **Summary phase-** review of key take home points, led by learners or educator

General Debriefing Goals:

- Create a safe learning environment
  - Share a learning contract (PreBrief)
  - Normalize gaps in performance
- Ask open ended questions (avoid yes/no questions)
- Try to facilitate the **team’s** discussion (avoid lecturing)

1. **Reactions Phase**

There are different perspectives on emotions and debriefing. One perspective: until emotions are addressed, it’s difficult for adult learners to “move on” to an analysis of their performance and opportunities for improvement. Another perspective is that adult learners should process their emotions independently.

Our perspective is the first. If a group or team member is emotionally charged (e.g. sad, mad or frustrated), it’s usually difficult for the individual or the group to be actively engaged, receptive to feedback and able to promote learning, until the emotions are addressed.

An example: a medication error occurs. One team member may think it is all their fault. They may feel embarrassed, judged, etc. If they can verbalize this, other team members may offer different perspectives, which enable the team to process the error together, potentially identifying contributing systems issues. If the emotions aren’t addressed- 3 separate people may feel embarrassed, responsible and not engage in a discussion, failing to identify systems issues which led to the error.

What you might say:

- “How did that feel?”
- “How did that go?”
- “Initial reactions?”
- “How are the rest of you feeling?”

1. Description phase

Summary of key events to ensure that educator and participants are on the same page. Summary are best elicited from learners, but facilitators should provide guidance and focus if there is not a shared understanding of the case.

What you might say:

- “Could someone summarize the case so we are all on the same page?”
- “From your perspective, what were the main issues you dealt with?”

1. **Analysis phase**

Promote reflection on performance (medical decision making, technical skills, teamwork and communication), identify opportunities for improvement. Facilitators may utilize both open ended questions allowing for learner self-assessment as well as more focused facilitation and direct feedback to ensure learning objectives are met. Typically, the authors utilize a combination of these strategies. The selection of debriefing strategies may be influenced by the time available for debriefing, the experience of the learners, and the experience of the facilitator.

What you might say:

- - - “Let’s talk more about the case.”
    - “What aspects did your team manage well? Why?”
    - “What could your team manage better next time? Why?”
    - “I want to spend a couple minutes talking about XXX. Can you tell me more about what was going on?”
    - I noticed you [*behavior*]…next time you may want to [*suggested behavior*]… because [*provide rationale*].

### 5) Summary phase

Opportunity to review key learning points. Participants’ or educator can identify take home points.

What you might say:

*Medical management/technical skills examples:*

- - - - “This scenario required a broad differential for central cyanosis and hypoxia in an infant including methemoglobinemia caused by lidocaine exposure.”
      - “This scenario is of an infant who presented cyanotic with oxygen saturations that did not improve with typical supportive measures, which is suggestive of decreased oxygen-hemoglobin dissociation and a symptom of methemoglobinemia.”
      - “When methemoglobinemia is suspected, the diagnosis can be confirmed with CO-oximetry.”
      - “The management of methemoglobinemia is primarily supportive unless the level is >20% in a symptomatic patient, then methylene blue can be considered as a treatment.”

*Teamwork/ communication examples:*

- - “Continued evaluation of patient and vital signs is necessary to assess the impact of interventions.”
  - "Designating leadership and team member roles are needed for coordinated team functioning."
  - "Roles should be assigned to specific individuals to avoid duplication/omission of tasks."
  - "Respect toward all team members is key to enable empowerment to speak up if patient safety issues arise."
  - "Use briefs or huddles to create a shared mental model for the working diagnosis and management plan."
  - "Closed-loop communication is of paramount importance to ensure safe and adequate communication."

**Debriefing Guide**

Below are examples of specific learning objective-based statements & questions you may use to debrief the team during the analysis phase.

- Perform a primary survey of a critically ill pediatric patient
- Implement a plan to stabilize a hypoxic and cyanotic neonate
- Develop a systematic approach for the evaluation of hypoxia and central cyanosis in a pediatric patient
- Describe the signs and symptoms of acquired methemoglobinemia in a pediatric patient
- Manage a pediatric patient with acquired methemoglobinemia
- Demonstrate teamwork and communication skills in a resuscitation setting

| **Examples of debriefing for different learning objectives** | | |
| --- | --- | --- |
| **Perform a primary survey of a critically ill pediatric patient** | | |
| Debriefer Script | Reference Material | Instructor Notes |
| “I noticed you [*quickly/took a while]* [*performed/to perform*] a complete primary survey. This was [*great/could have been problematic]* since the primary survey quickly identifies life threatening conditions.”   - “What [helped/hindered] you?” - “What could you do differently?” | Primary survey goals:   - Airway, Breathing, Circulation, Disability (e.g. pupils, Glasgow Coma Scale) and Exposure (remove clothing, temperature check and control) |  |
| **Implement a plan to stabilize a neonate with evidence of central cyanosis and hypoxia** | | |
| Debriefer Script | Reference Material | Instructor Notes |
| “I noticed you *[quickly/took a while]* [*identified/to identify*] the child was hypoxic with the physical exam finding of cyanosis. This was [*great/could be problematic*] because a child presenting with these symptoms requires immediate interventions to support their breathing and oxygenation.”   - “What [*helped/hindered*] you to [*recognize/treat*] it?” - “I noticed you [quickly/took a while] to intervene after you identified cyanosis and hypoxia. This was [great/problematic] because I was concerned persistent hypoxia and respiratory distress could lead to further clinical deterioration. - “How did you decide which interventions to try?” - “What other options could you have tried?” | Concerning physical exam findings:   - Increased work of breathing - Central cyanosis (seen by darker color of lips or bluish coloring of perioral skin/mucosal membranes) - Low peripheral oxygen saturation noted on pulse oximetry   Recognition of central cyanosis:   - If using a high-tech simulator, this can be programmed into the simulation. - Simulators of varying skin color should be used so participants can understand how cyanosis presents in children with varying pigmentation of their skin. - If using a low-tech simulator, this can be done by stating that the child has a bluish, gray, or white discoloration of their lips. |  |

| **Develop a systematic approach for the evaluation of central cyanosis and hypoxia in a pediatric patient** | | |
| --- | --- | --- |
| Debriefer Script | Reference Material | Instructor Notes |
| “I noticed you [did/did not] discuss a differential for central cyanosis and hypoxia in a neonate. I was wondering [how you were thinking through possibilities/what diagnoses you were considering]. How did you come to this differential?”   - “What did other team members identify as the differential/the working diagnosis/differential?” - “Was anyone concerned that something else might be going on? Why?” | Potential causes of central cyanosis and hypoxia in a neonate:   - Cyanotic heart disease - Pulmonary disease - Anatomic airway anomalies - Sepsis - Methemoglobinemia |  |

| **Describe the signs and symptoms of acquired methemoglobinemia in a pediatric patient** | | |
| --- | --- | --- |
| Debriefer Script | Reference Material | Instructor Notes |
| “I noticed you *[quickly/took a while] [identified/to identify]* that oxygen or additional ventilator support did not increase peripheral oxygen saturations. This was *[great/could be problematic]* because this will *[allow for quick/delay]* identification of the diagnosis.   - What other signs can you look for that will guide you toward a diagnosis of methemoglobinemia? | Recognition of methemoglobinemia:   - Peripheral oxygen saturations will not improve with supplemental oxygen - Arterial oxygen saturations on arterial blood gas are >80mmHg - Blood remains dark when exposed to oxygen during phlebotomy   Diagnosis of methemoglobinemia:   - CO-oximetry - Methemoglobin level |  |
| **Manage a pediatric patient with acquired methemoglobinemia** | | |
| Debriefer Script | Reference Material | Instructor Notes |
| “I noticed you [*identified*/*did not identify*] a plan of care to manage the patient’s methemoglobinemia. This was [*great/could be problematic*] because the patient may require treatment urgently.   - How do you manage acute, symptomatic, acquired methemoglobinemia? | Treatment of methemoglobinemia:   - Methemoglobin level less than 20% and asymptomatic: provide supportive care - Methemoglobin level >20% and/or symptomatic: consider use of methylene blue |  |

| **Examples for debriefing different Teamwork Learning Objectives** | | | |
| --- | --- | --- | --- |
| **Roles and Responsibilities** | | | |
| Debriefer Script | Reference Material | | Instructor Notes |
| “From my perspective, it looked like you (*did/did not) have* clear team roles. I think this is (*great/concerning)* because clear team roles can help a team function smoothly, improving how quickly interventions take place and reducing errors.” | Team leader   - Clear direction, coordination, timely interventions - Stands at foot of patient with hands off of patient so can see the big picture   Airway/Procedure MD   - Manage airway - Head of patient   Survey MD   - Primary, Secondary survey, pulses with CPR, reassess   Nursing roles   - Medication Prep (draw-up meds) - Medication Admin (give meds) - Documenting (time keeper) | |  |
| **Closed-loop communication** | | | |
| Debriefer Script | | Reference Material | Instructor Notes |
| “I noticed that your team *(did/didn’t/took a while to)* (*brief* *prior to the initial patient assessment/huddle after the initial evaluation).* I thought this (*was* *great/could have helped to*) facilitate patient care.”   - “What *(helped/hindered)* your team from (*briefing/huddling*)?” - “How did that impact your team?” - “What could your team have done differently?” - “How can you make sure that *(does/doesn’t*) happen again?” | | The goal of a brief/huddle is to create a shared mental model. Assure all team members know what the working diagnosis is, management priorities and next steps in care.   - Everyone on the team is responsible for making this happen. Anyone can ask for a brief/huddle. Brief/huddle is usually led by team leader. - If one team member doesn’t know what’s up or what’s next- s/he is probably not alone. |  |

| **Directed call out** | | |
| --- | --- | --- |
| Debriefer Script | Reference Material | Instructor Notes |
| “I noticed that you (*did/didn’t/intermittently*) used (*people's names/roles/eye contact*) when (*calling out orders/asking for assistance*). I thought this was (*great/could have been more directed*) in order to facilitate communication.”   - “What did you notice about orders/questions that were asked?” - “How did this impact your team?” | Directed call out. A tactical communication skill to assure that important orders/questions are specifically directed to one individual (rather than called out into the air).  Example:   - “Lucy-What’s the oxygen saturation?” - “Team leader- His oxygen saturation is not improving with oxygen supplementation.” |  |
| **Closed loop communication/Check back** | | |
| Debriefer Script | Reference Material | Instructor Notes |
| “I noticed that you used closed-loop communication *(consistently/rarely)*. Closed-loop communication can be critical for catching errors and assuring that *(information/an order/a request)* is heard.”   - “Tell me about your communication loops” - “How did that impact your team?” - “Has anyone seen problems with this in a patient resuscitation? Can you tell us about that?” - “Has anyone seen closed loop communication prevent an error?” - “How could you do it differently next time?” | Closed loop communication/check back is a strategy that requires verification of information. This enables the sender of the message to verify it has been heard and heard correctly. It enables the receiver to confirm what they heard is correct.   - Team leader “Float nurse, call for portable x-ray.” - Float nurse “Calling technician for a portable x-ray.” - Team leader “Correct” |  |

**Lidocaine-Induced Methemoglobinemia Medical Management Evaluation/Debriefing Form**

This checklist identifies core medical management /technical skills. It is hard to discuss more than 2-3 of these during one debriefing session.

**Performing a primary survey □** Done Well  **□** Needs Work

Specific comments: _____________________________________________________________________________

_____________________________________________________________________________________________

*Discussion Points: What did you think of the timeliness/completeness of the ABCDE’s (airway, breathing, circulation, disability, exposure)? What could you do differently?*

______________________________________________________________________________

**Stabilize a neonate with central cyanosis and hypoxia** □ Done Well □ Needs Work

Specific comments: _____________________________________________________________________________

_____________________________________________________________________________________________

_____________________________________________________________________________________________

*Discuss Points: How can you assist oxygenation in neonates? (nasal cannula, face masks, with variable delivery of fiO2, non-invasive positive pressure, intubation)*

**Evaluate central cyanosis and hypoxia in a neonate** *□* Done Well □ Needs Work

Specific comments: _____________________________________________________________________________

________*________________________________________________________________________________________________________________________________________________________________________*_________*_*

*Discuss Points: What etiologies can cause hypoxia in a neonate and how would you evaluate these? (pulmonary disease: exam, consider chest radiograph; congenital heart disease (exam, consider chest radiograph, ECG, echocardiogram); sepsis: CBC, UA, LP, blood/urine/CSF cultures; airway abnormality: exam, advanced diagnostics like bronchoscopy or laryngoscopy; methemoglobinemia: co-oximetry)*

**Recognize signs and symptoms of methemoglobinemia □** Done Well **□** Needs Work

Specific comments: _____________________________________________________________________________

_____________________________________________________________________________________________

_____________________________________________________________________________________________

*Discuss Points: What types of exposures can cause methemoglobinemia in children? What are symptoms, peripheral oxygen saturations, and laboratory values concerning for a diagnosis of methemoglobinemia?*

**Management of methemoglobinemia □** Done Well **□** Needs Work

Specific comments: _____________________________________________________________________________

_____________________________________________________________________________________________

_____________________________________________________________________________________________

*Discussion Points: How do you manage methemoglobinemia and when would you use methylene blue to treat methemoglobinemia? (supportive measures including oxygen, methylene blue when level >20-30% and patient is symptomatic)*

**Lidocaine-Induced Methemoglobinemia Teamwork and Communication Evaluation**

This checklist identifies core medical management /technical skills. It is hard to discuss more than 2-3 of these during one debriefing session.

**Leader/Roles Identified & Maintained □** Done Well **□** Needs Work

Specific comments: _____________________________________________________________________________

_____________________________________________________________________________________________

_____________________________________________________________________________________________

*Discussion Points: What helped/hindered having clear leadership and roles?*

**Directed Call out □** Done Well **□** Needs Work

Specific comments: _____________________________________________________________________________

_____________________________________________________________________________________________

_____________________________________________________________________________________________

*Discussion Points: How were orders given- e.g. “Into the air” or directed at specific individuals? How did that impact you? How could they be delivered more effectively?*

**Check back/Closed loop communication □** Done Well **□** Needs Work

Specific comments: _____________________________________________________________________________

_____________________________________________________________________________________________

_____________________________________________________________________________________________

*Discussion Points: Describe closed loop communication.*

**Shared Mental Model □** Done Well **□** Needs Work

Specific comments: _____________________________________________________________________________

_____________________________________________________________________________________________

_____________________________________________________________________________________________

*Discussion Points: How did team members share information/working diagnosis/management plan ((brief/huddle)?*

**References**

1. Eppich W, Cheng A. Promoting Excellence and Reflective Learning in Simulation (PEARLS): Development and Rationale for a Blended Approach to Healthcare Simulation Debriefing
