# Supplementary material for: Pediatric Toxidrome Simulation Curriculum: Lidocaine-Induced Methemoglobinemia
Source: MedEdPORTAL. 2021 Jan 28;17:11089. doi: 10.15766/mep_2374-8265.11089 (PMC7842087; doi:10.15766/mep_2374-8265.11089)
Supplement: Supplementary file 1 — Simulation Case.docxEnvironment Preparation.docxImages.pptxTeamwork and Communication Glossary.docxDebriefing Guide.docxEvaluation Form.docxDidactics.pptx [file mep_2374-8265.11089-s001.zip › F. Evaluation Form.docx]

**Appendix F: Methemoglobinemia Simulation Evaluation Form**

**Instructor:** __________________ **Date:**  ____________ **Role**: _______________

|  | Strongly  Disagree | Disagree | Neutral | Agree | Strongly  Agree |
| --- | --- | --- | --- | --- | --- |
| This case presented during the simulation is relevant to my work. | 1 | 2 | 3 | 4 | 5 |
| The simulation case was realistic. | 1 | 2 | 3 | 4 | 5 |
| This simulation case was effective in teaching basic resuscitation skills. | 1 | 2 | 3 | 4 | 5 |
| The debrief promoted reflection and team discussion. | 1 | 2 | 3 | 4 | 5 |
| The facilitators created a safe environment for discussion and exploration. | 1 | 2 | 3 | 4 | 5 |

After participating in this session how confident are you in your ability to:

|  | Very Unconfident | Unconfident | Neutral | Confident | Very Confident |
| --- | --- | --- | --- | --- | --- |
| Perform a primary survey of a critically ill pediatric patient | 1 | 2 | 3 | 4 | 5 |
| Implement a plan to stabilize a neonate with evidence of central cyanosis and hypoxia | 1 | 2 | 3 | 4 | 5 |
| Develop a systematic approach for the evaluation of central cyanosis and hypoxia in a pediatric patient | 1 | 2 | 3 | 4 | 5 |
| Describe the signs and symptoms of acquired methemoglobinemia in a pediatric patient | 1 | 2 | 3 | 4 | 5 |
| Manage a pediatric patient with acquired methemoglobinemia | 1 | 2 | 3 | 4 | 5 |
| Demonstrate effective teamwork and communication skills. | 1 | 2 | 3 | 4 | 5 |

Can you list/describe 1 or more ways this session will change how you do your job?

How could we improve this simulation?

Additional Comments:
