# Supplementary material for: Pediatric Toxidrome Simulation Curriculum: Lidocaine-Induced Methemoglobinemia
Source: MedEdPORTAL. 2021 Jan 28;17:11089. doi: 10.15766/mep_2374-8265.11089 (PMC7842087; doi:10.15766/mep_2374-8265.11089)
Supplement: Supplementary file 1 — Simulation Case.docxEnvironment Preparation.docxImages.pptxTeamwork and Communication Glossary.docxDebriefing Guide.docxEvaluation Form.docxDidactics.pptx [file mep_2374-8265.11089-s001.zip › G. Didactics.pptx]

## Slide 1
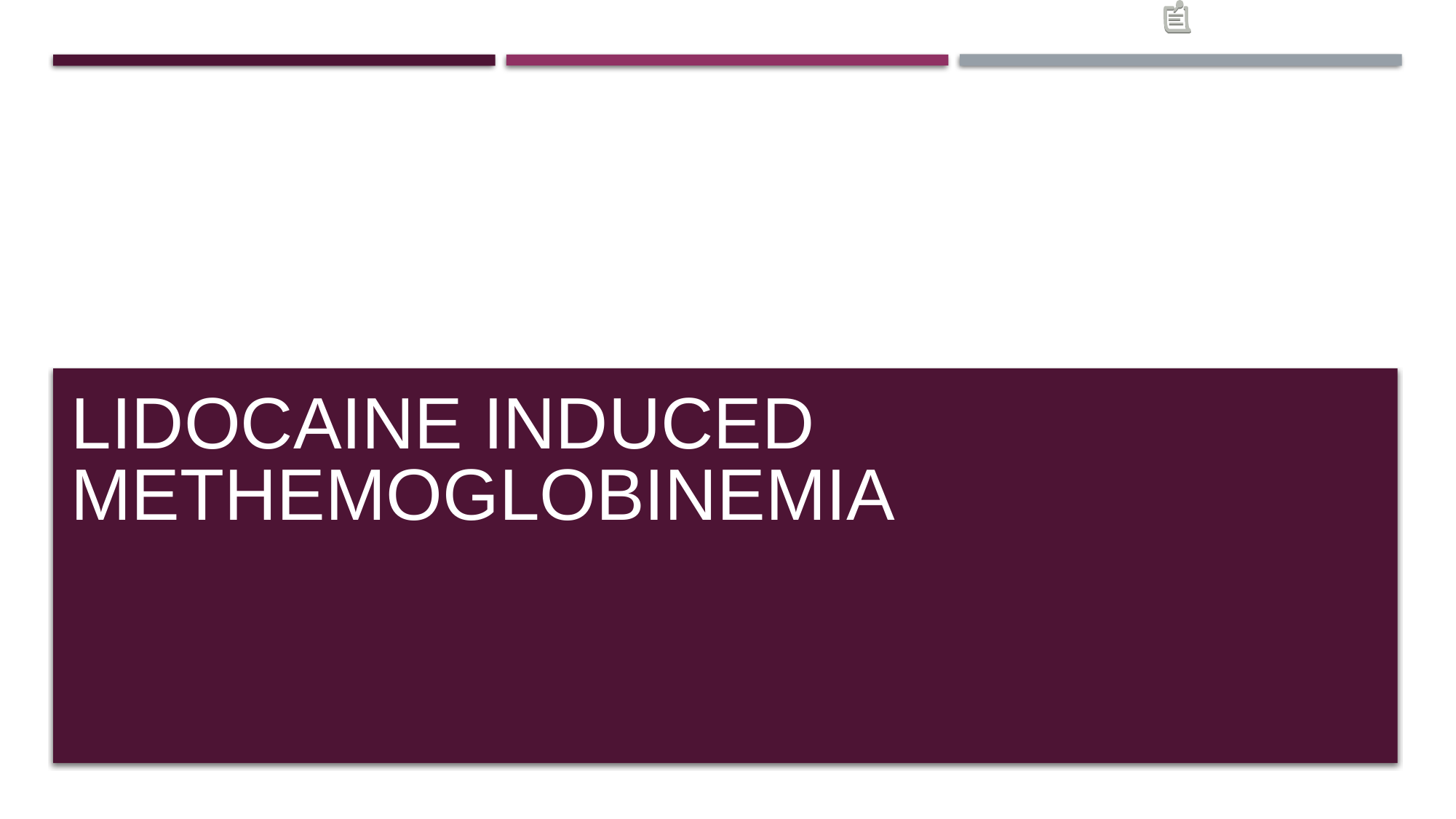

Lidocaine Induced
Methemoglobinemia

## Slide 2
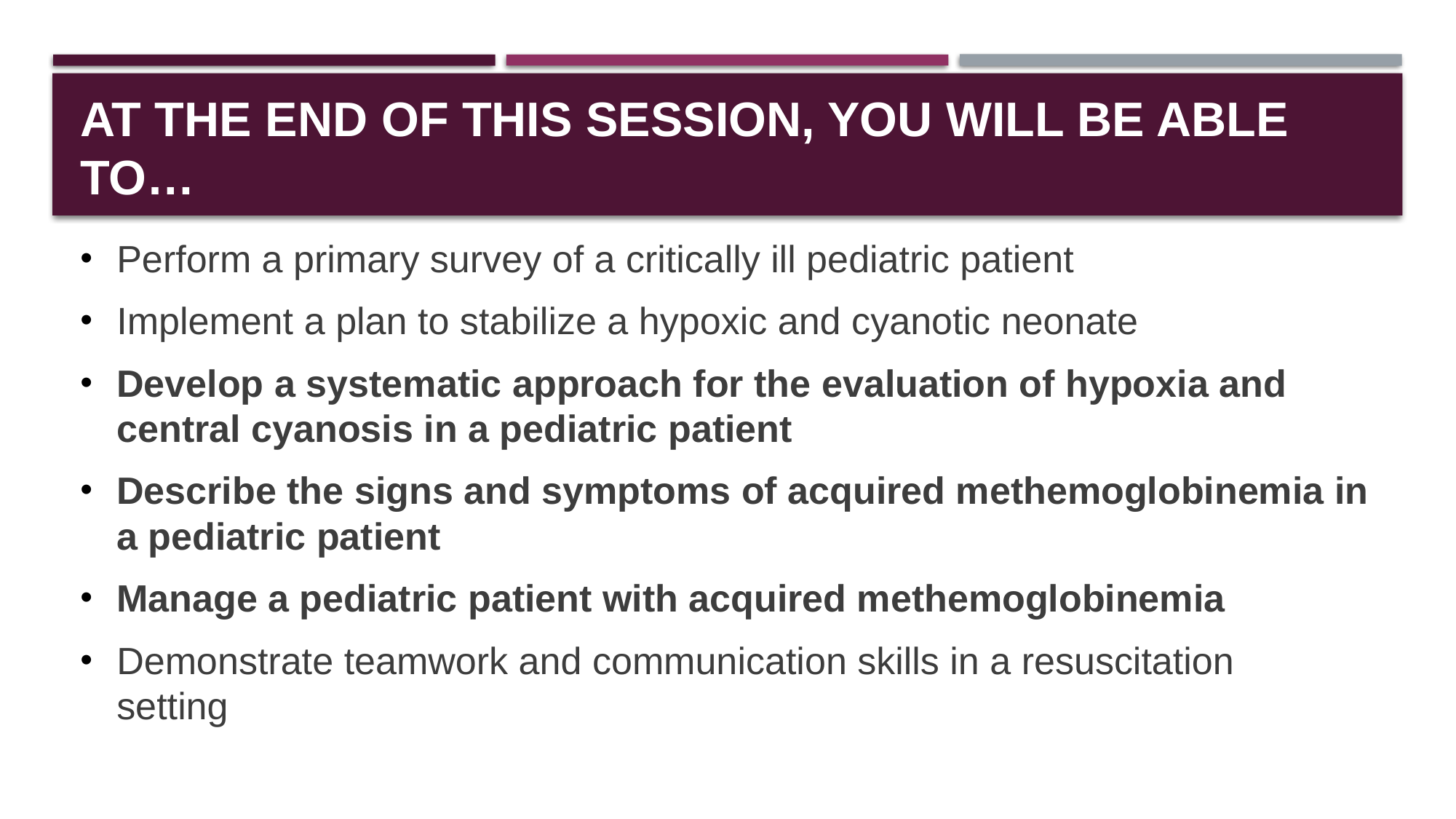

# At the end of this session, you will be able to…
Perform a primary survey of a critically ill pediatric patient
Implement a plan to stabilize a hypoxic and cyanotic neonate
Develop a systematic approach for the evaluation of hypoxia and central cyanosis in a pediatric patient
Describe the signs and symptoms of acquired methemoglobinemia in a pediatric patient
Manage a pediatric patient with acquired methemoglobinemia
Demonstrate teamwork and communication skills in a resuscitation setting

## Slide 3
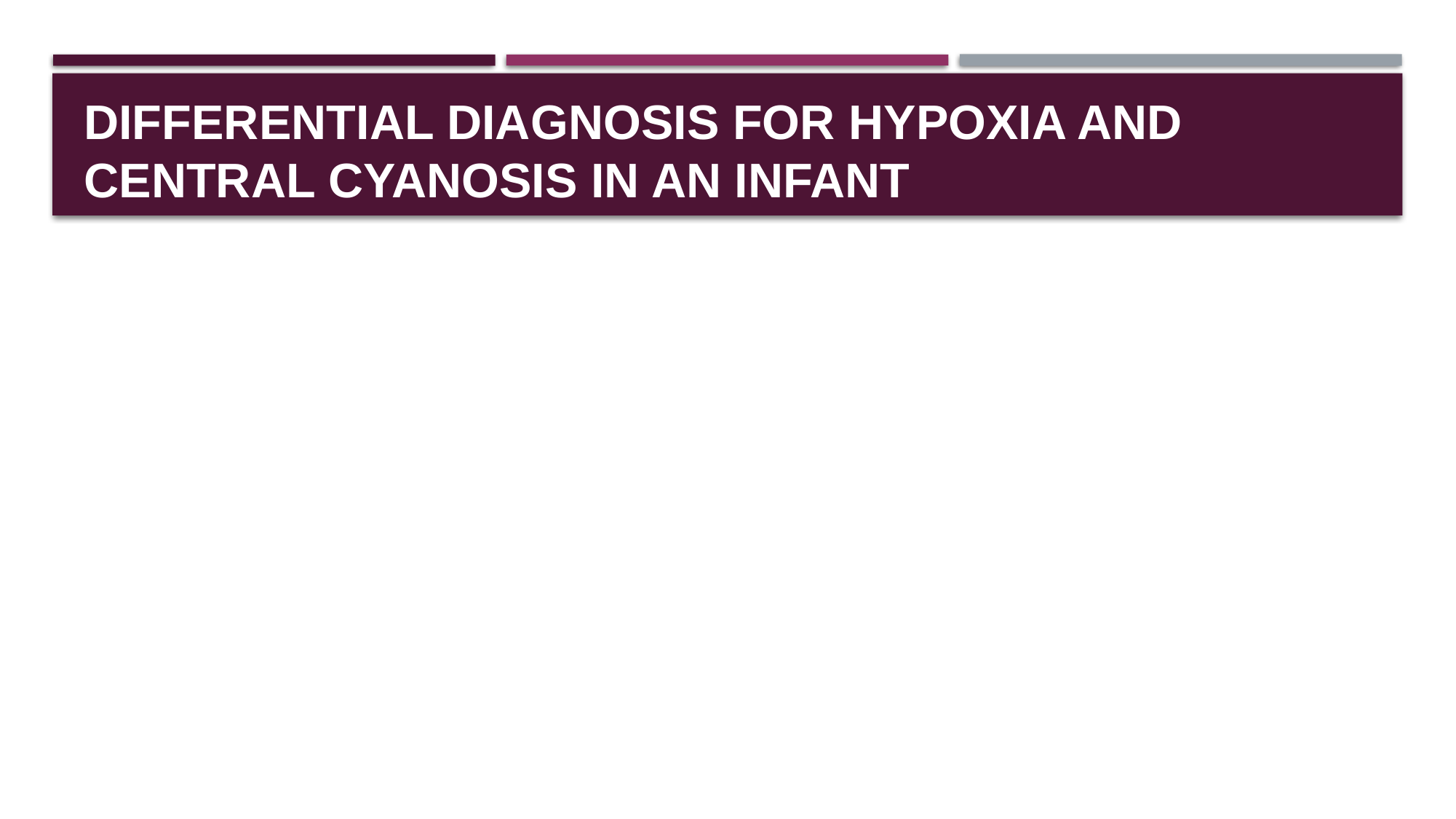

# Differential diagnosis for hypoxia and central cyanosis in an infant

## Slide 4
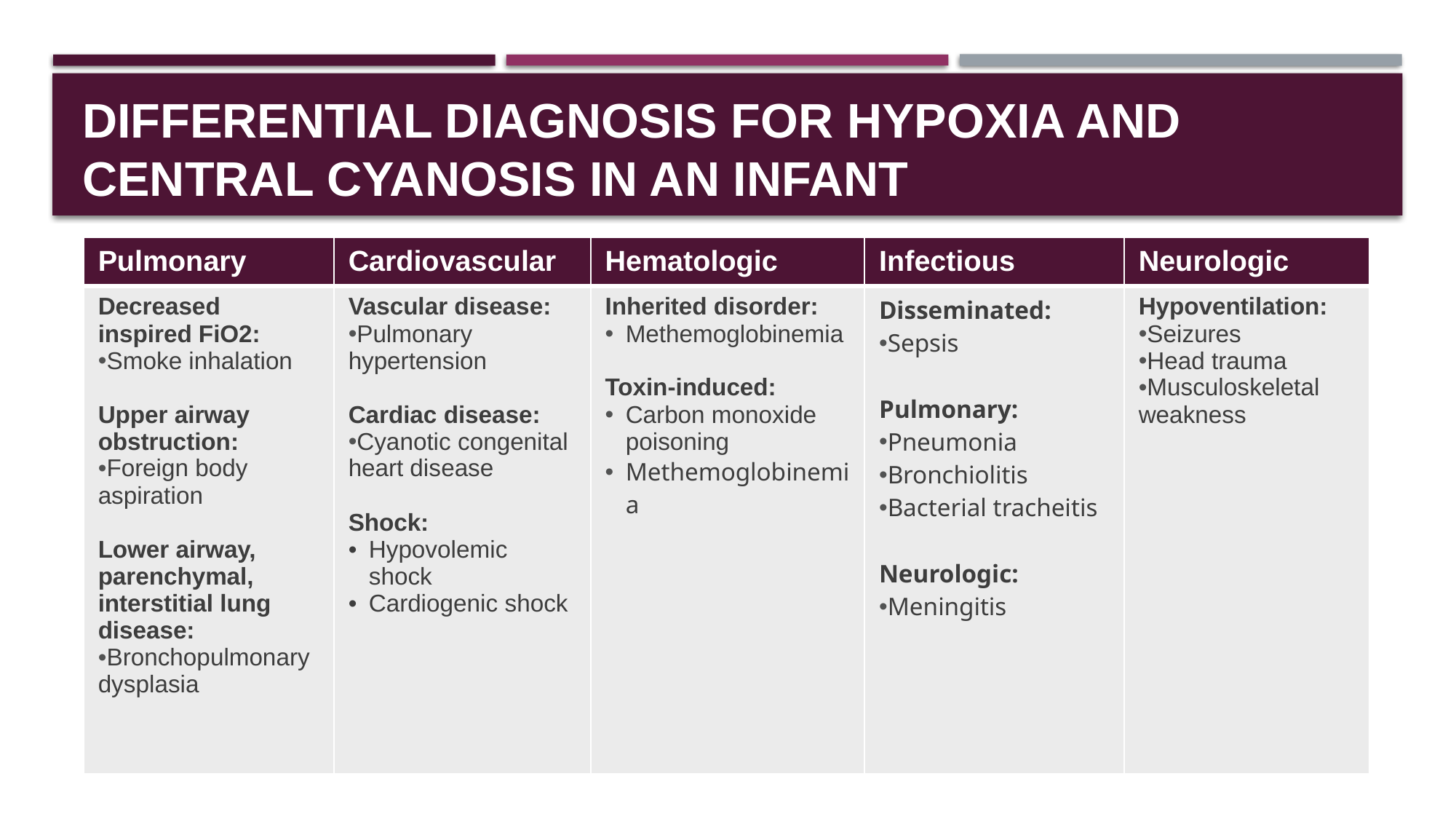

Differential diagnosis for Hypoxia and central cyanosis in an infant
| Pulmonary | Cardiovascular | Hematologic | Infectious | Neurologic |
| --- | --- | --- | --- | --- |
| Decreased inspired FiO2: Smoke inhalation Upper airway obstruction: Foreign body aspiration Lower airway, parenchymal, interstitial lung disease: Bronchopulmonary dysplasia | Vascular disease: Pulmonary hypertension Cardiac disease: Cyanotic congenital heart disease Shock: Hypovolemic shock Cardiogenic shock | Inherited disorder: Methemoglobinemia Toxin-induced: Carbon monoxide poisoning Methemoglobinemia | Disseminated: Sepsis Pulmonary: Pneumonia Bronchiolitis Bacterial tracheitis Neurologic: Meningitis | Hypoventilation: Seizures Head trauma Musculoskeletal weakness |

## Slide 5
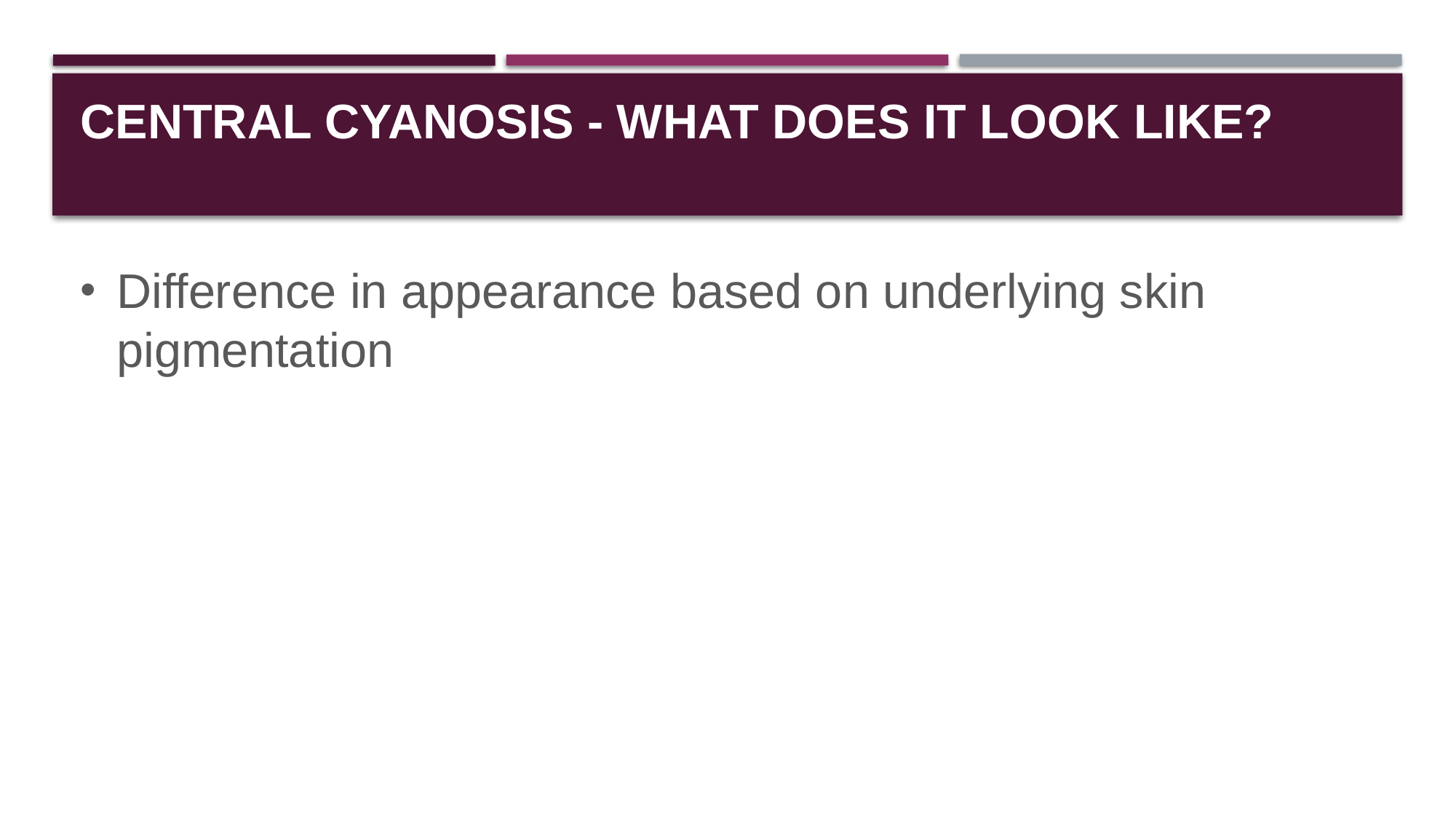

# Central Cyanosis - What does it look like?
Difference in appearance based on underlying skin pigmentation

## Slide 6
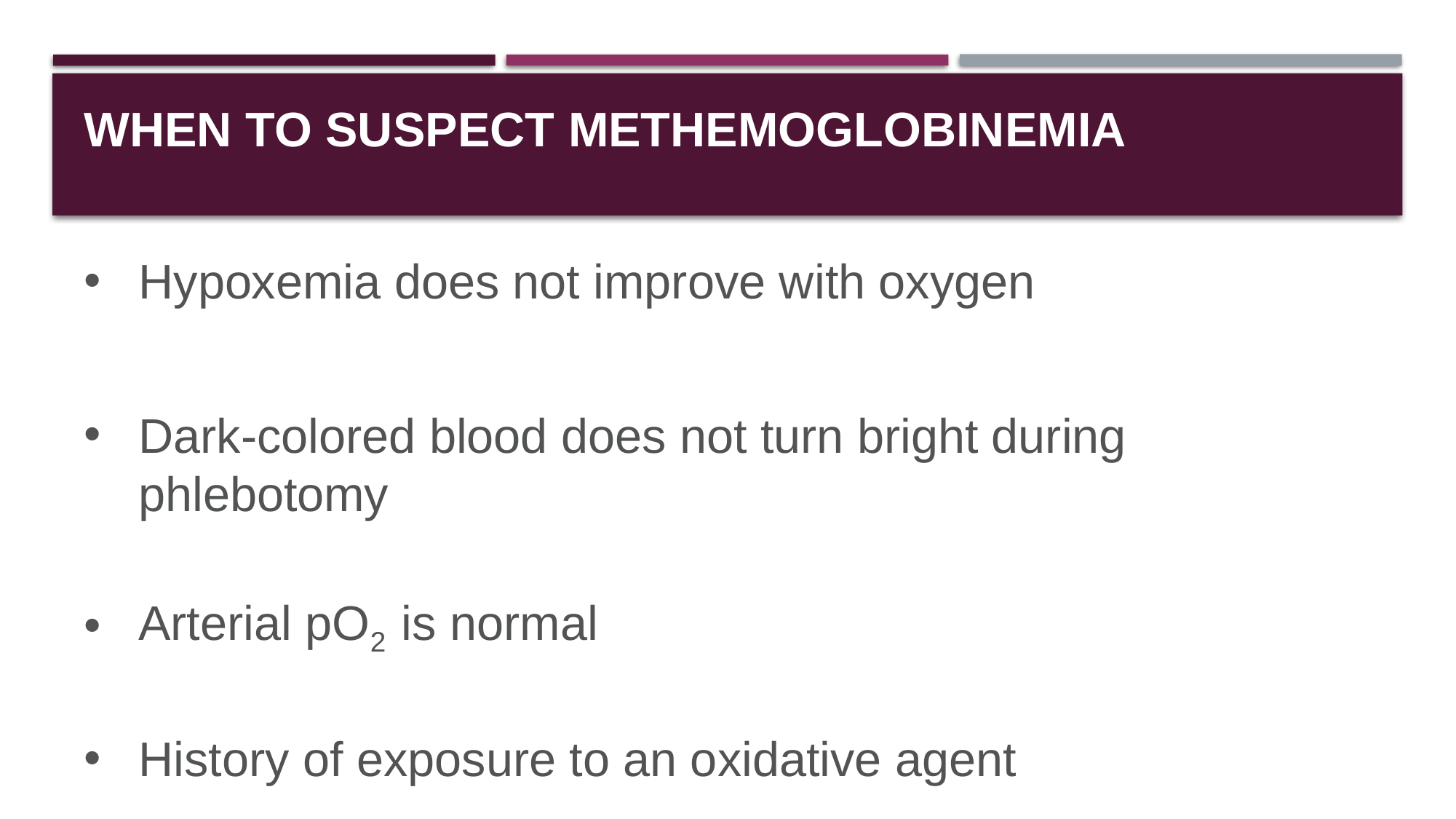

# When to suspect methemoglobinemia
Hypoxemia does not improve with oxygen
Dark-colored blood does not turn bright during phlebotomy
Arterial pO2 is normal
History of exposure to an oxidative agent

## Slide 7
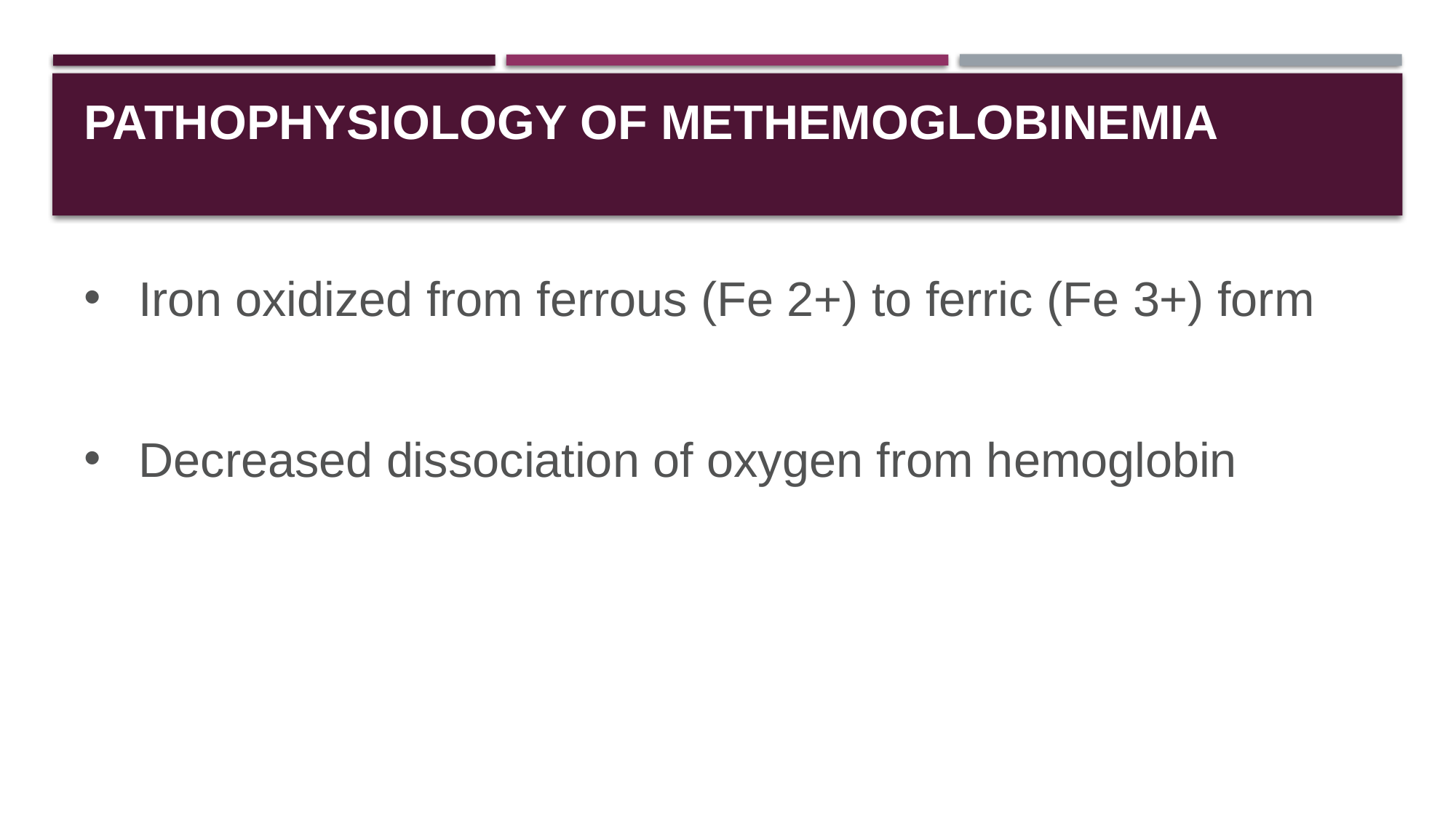

# Pathophysiology of methemoglobinemia
Iron oxidized from ferrous (Fe 2+) to ferric (Fe 3+) form
Decreased dissociation of oxygen from hemoglobin

## Slide 8
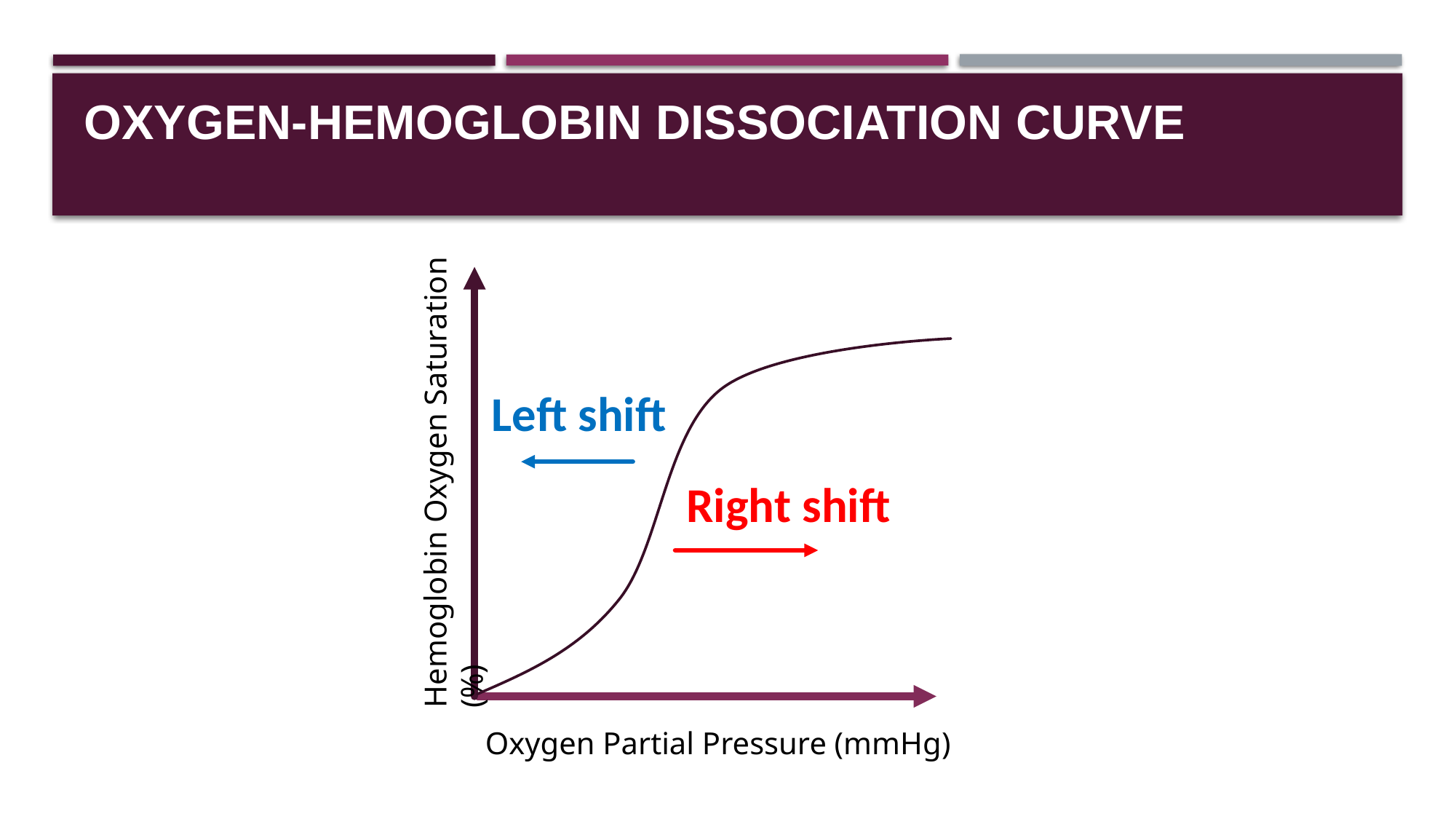

# Oxygen-hemoglobin dissociation curve
Left shift
Hemoglobin Oxygen Saturation (%)
Right shift
Oxygen Partial Pressure (mmHg)

## Slide 9
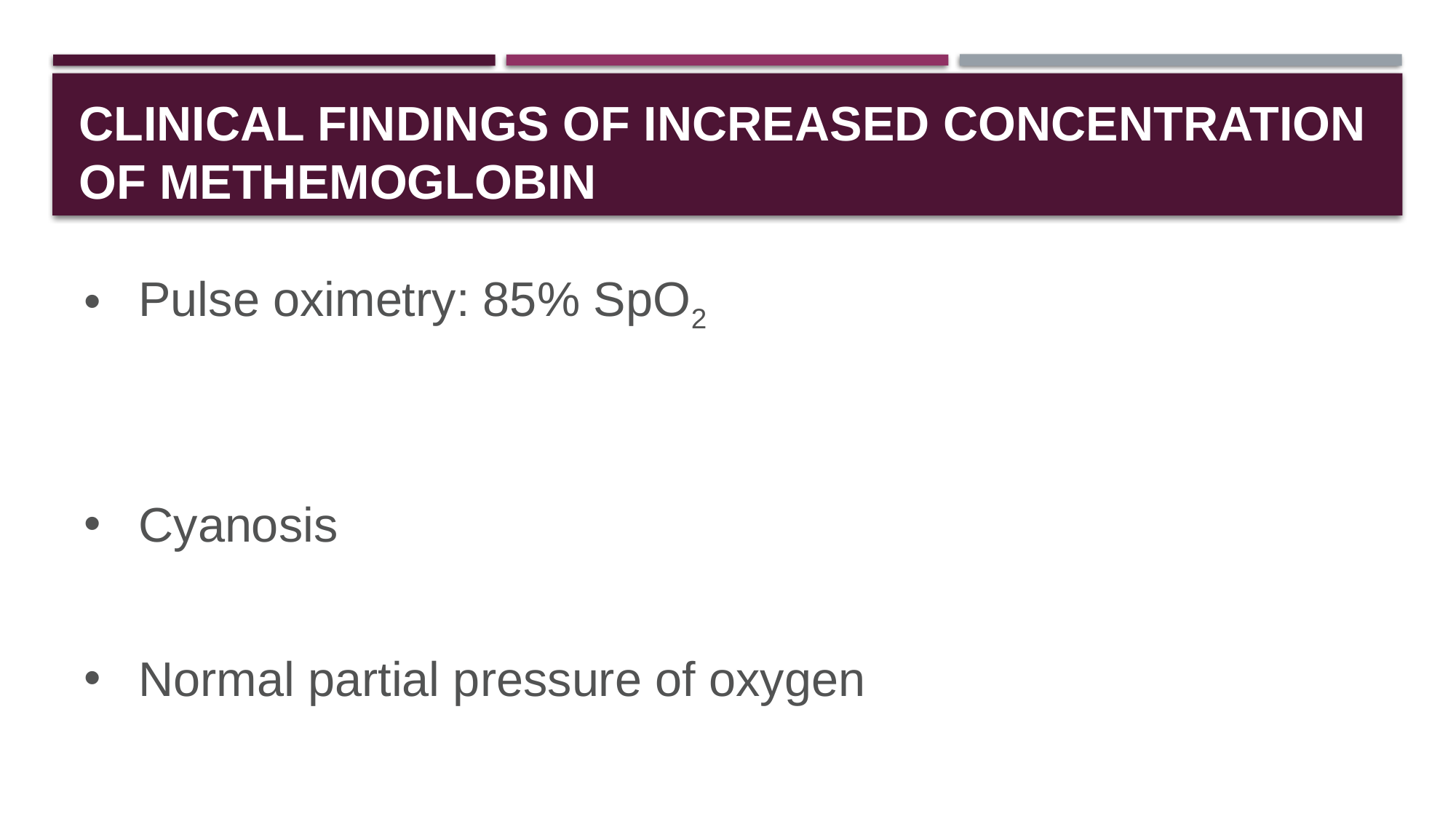

# Clinical findings of increased concentration of methemoglobin
Pulse oximetry: 85% SpO2
Cyanosis
Normal partial pressure of oxygen

## Slide 10
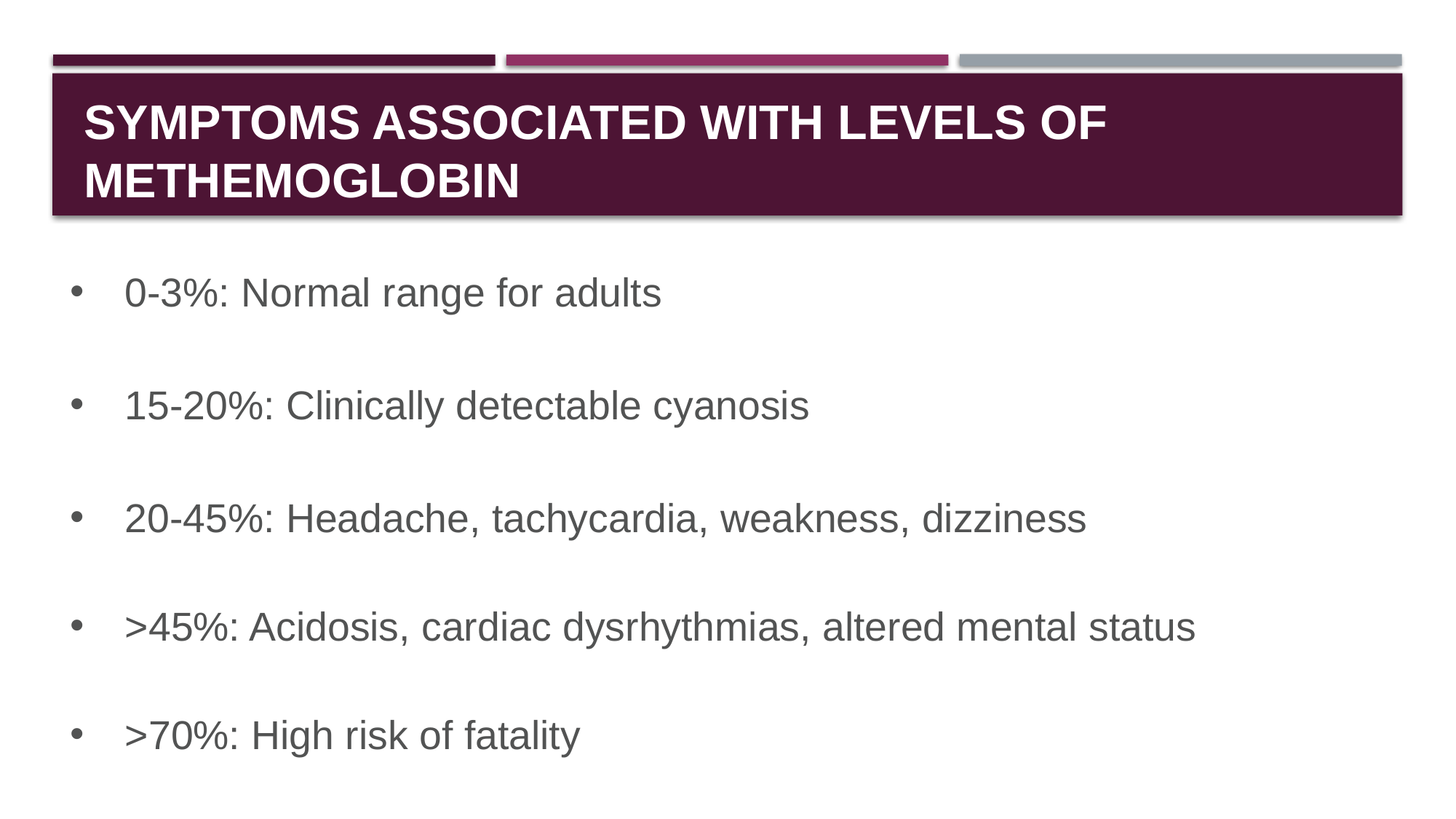

# Symptoms associated with levels of methemoglobin
0-3%: Normal range for adults
15-20%: Clinically detectable cyanosis
20-45%: Headache, tachycardia, weakness, dizziness
>45%: Acidosis, cardiac dysrhythmias, altered mental status
>70%: High risk of fatality

## Slide 11
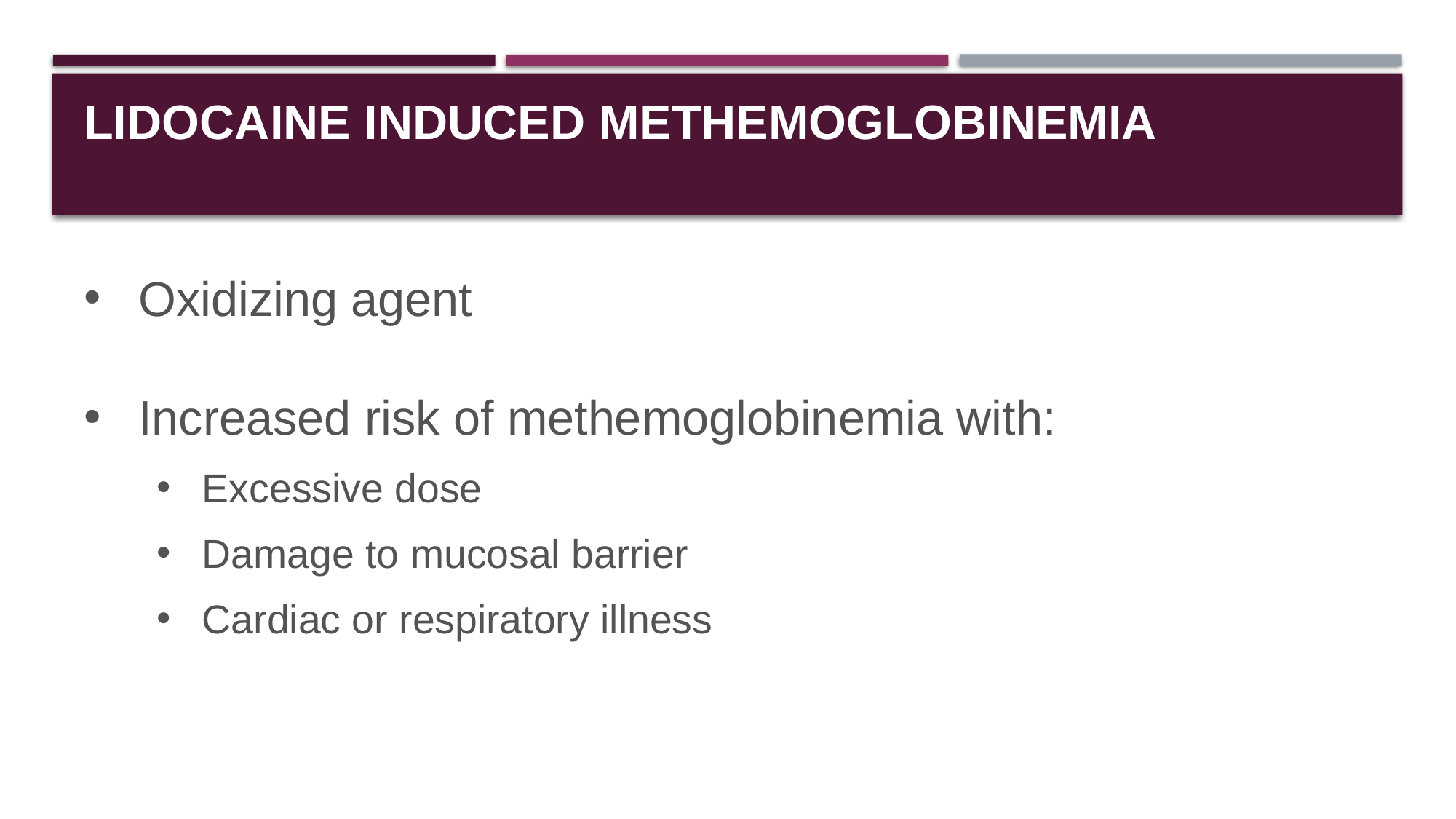

# Lidocaine induced methemoglobinemia
Oxidizing agent
Increased risk of methemoglobinemia with:
Excessive dose
Damage to mucosal barrier
Cardiac or respiratory illness

## Slide 12
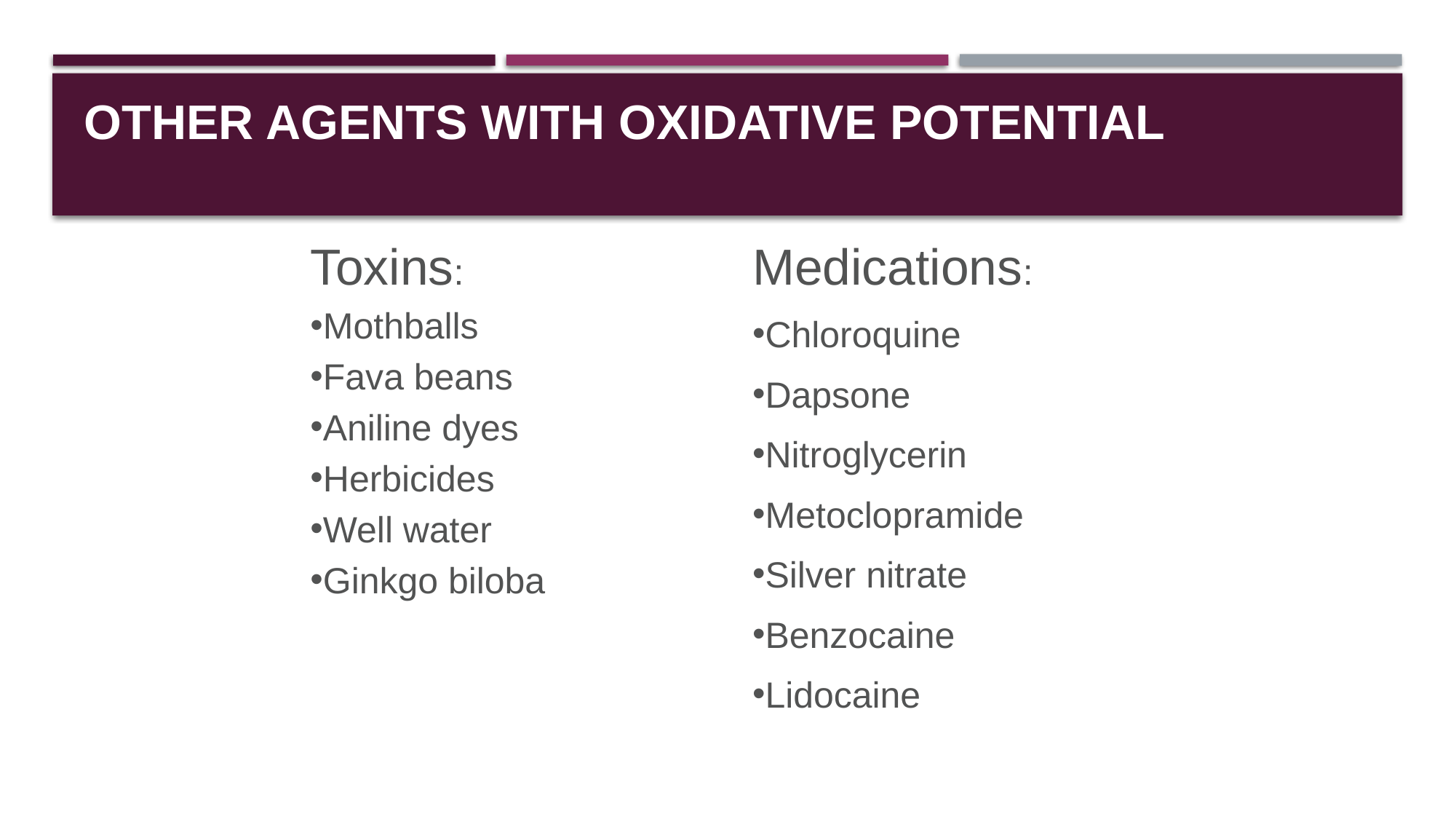

# Other agents with oxidative potential
Medications:
Chloroquine
Dapsone
Nitroglycerin
Metoclopramide
Silver nitrate
Benzocaine
Lidocaine
Toxins:
Mothballs
Fava beans
Aniline dyes
Herbicides
Well water
Ginkgo biloba

## Slide 13
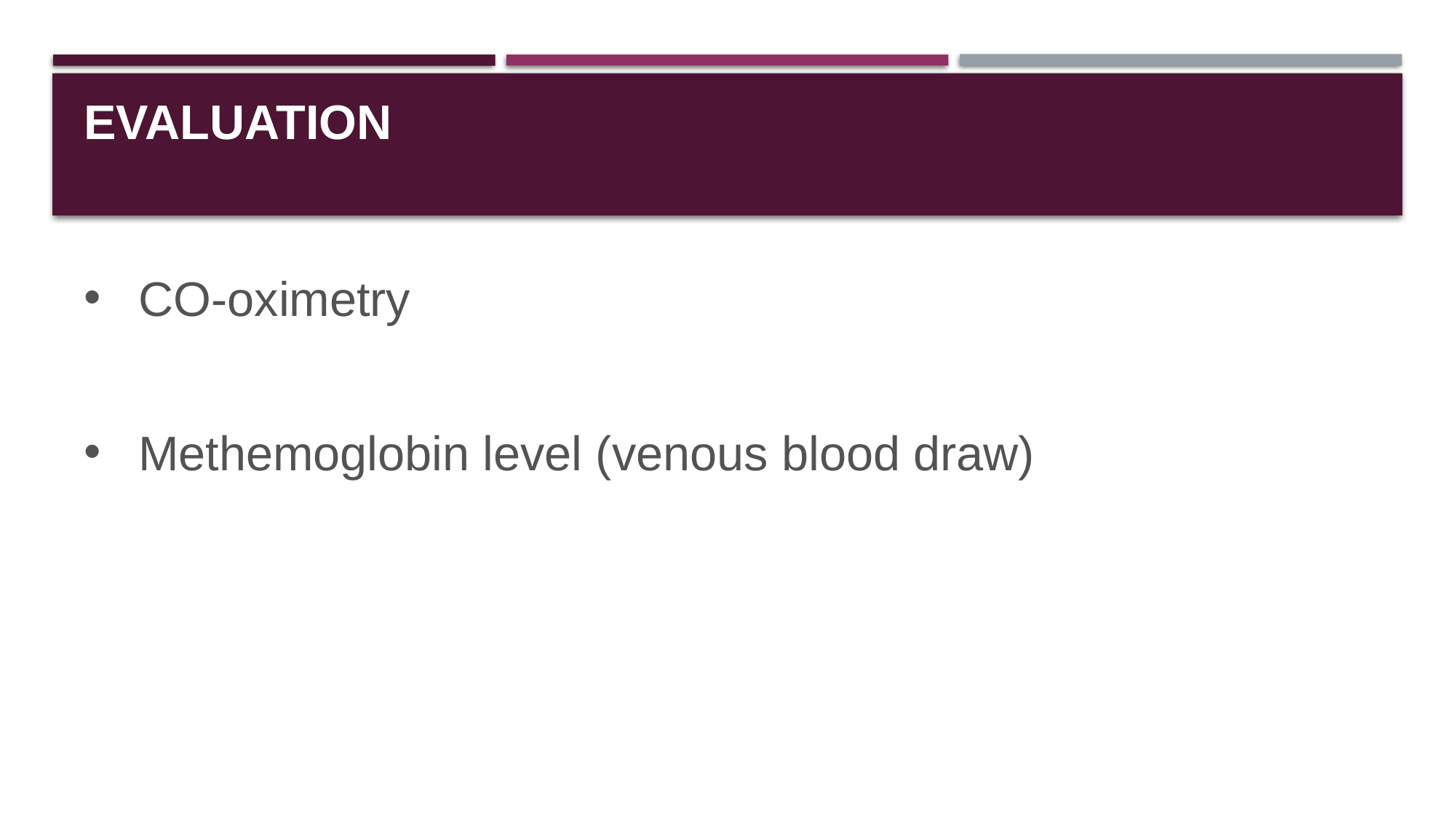

# Evaluation
CO-oximetry
Methemoglobin level (venous blood draw)

## Slide 14
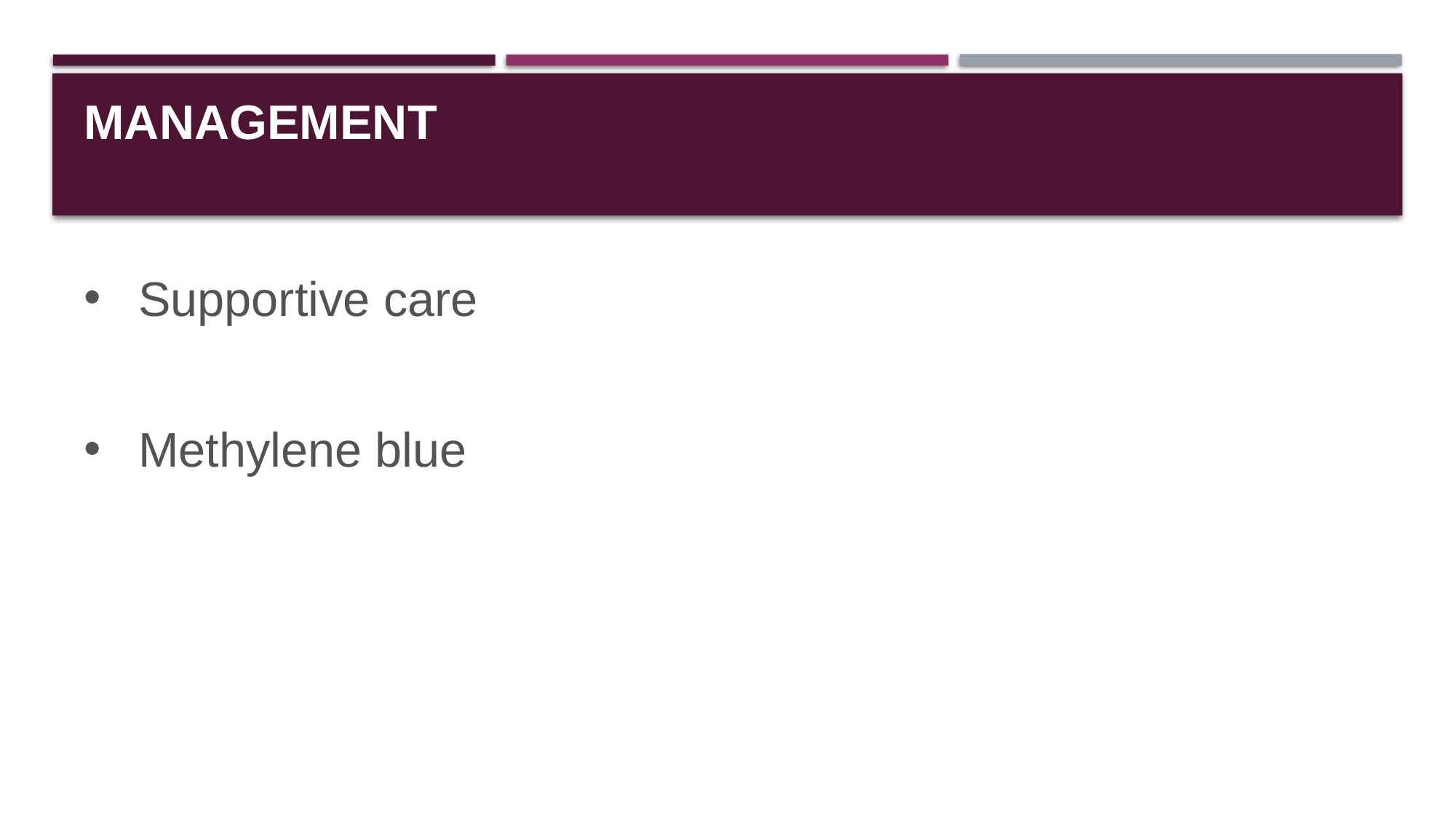

# Management
Supportive care
Methylene blue

## Slide 15
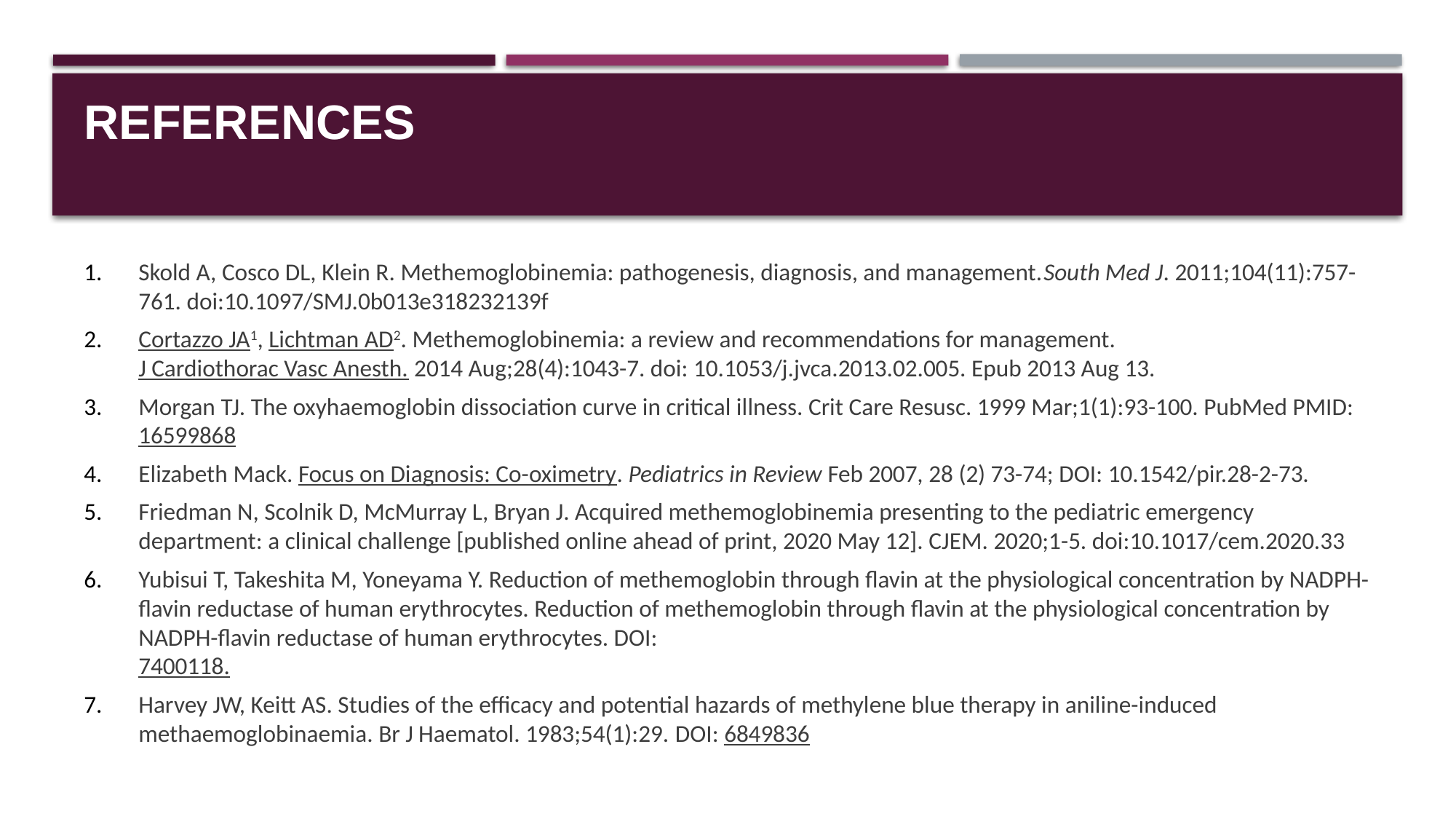

# References
Skold A, Cosco DL, Klein R. Methemoglobinemia: pathogenesis, diagnosis, and management.South Med J. 2011;104(11):757-761. doi:10.1097/SMJ.0b013e318232139f
Cortazzo JA1, Lichtman AD2. Methemoglobinemia: a review and recommendations for management. J Cardiothorac Vasc Anesth. 2014 Aug;28(4):1043-7. doi: 10.1053/j.jvca.2013.02.005. Epub 2013 Aug 13.
Morgan TJ. The oxyhaemoglobin dissociation curve in critical illness. Crit Care Resusc. 1999 Mar;1(1):93-100. PubMed PMID: 16599868
Elizabeth Mack. Focus on Diagnosis: Co-oximetry. Pediatrics in Review Feb 2007, 28 (2) 73-74; DOI: 10.1542/pir.28-2-73.
Friedman N, Scolnik D, McMurray L, Bryan J. Acquired methemoglobinemia presenting to the pediatric emergency department: a clinical challenge [published online ahead of print, 2020 May 12]. CJEM. 2020;1-5. doi:10.1017/cem.2020.33
Yubisui T, Takeshita M, Yoneyama Y. Reduction of methemoglobin through flavin at the physiological concentration by NADPH-flavin reductase of human erythrocytes. Reduction of methemoglobin through flavin at the physiological concentration by NADPH-flavin reductase of human erythrocytes. DOI:
7400118.
Harvey JW, Keitt AS. Studies of the efficacy and potential hazards of methylene blue therapy in aniline-induced methaemoglobinaemia. Br J Haematol. 1983;54(1):29. DOI: 6849836
