# Supplementary figures and images for: Pediatric Toxidrome Simulation Curriculum: Lidocaine-Induced Methemoglobinemia
Source: MedEdPORTAL. 2021 Jan 28;17:11089. doi: 10.15766/mep_2374-8265.11089 (PMC7842087; doi:10.15766/mep_2374-8265.11089)

## Slide 1
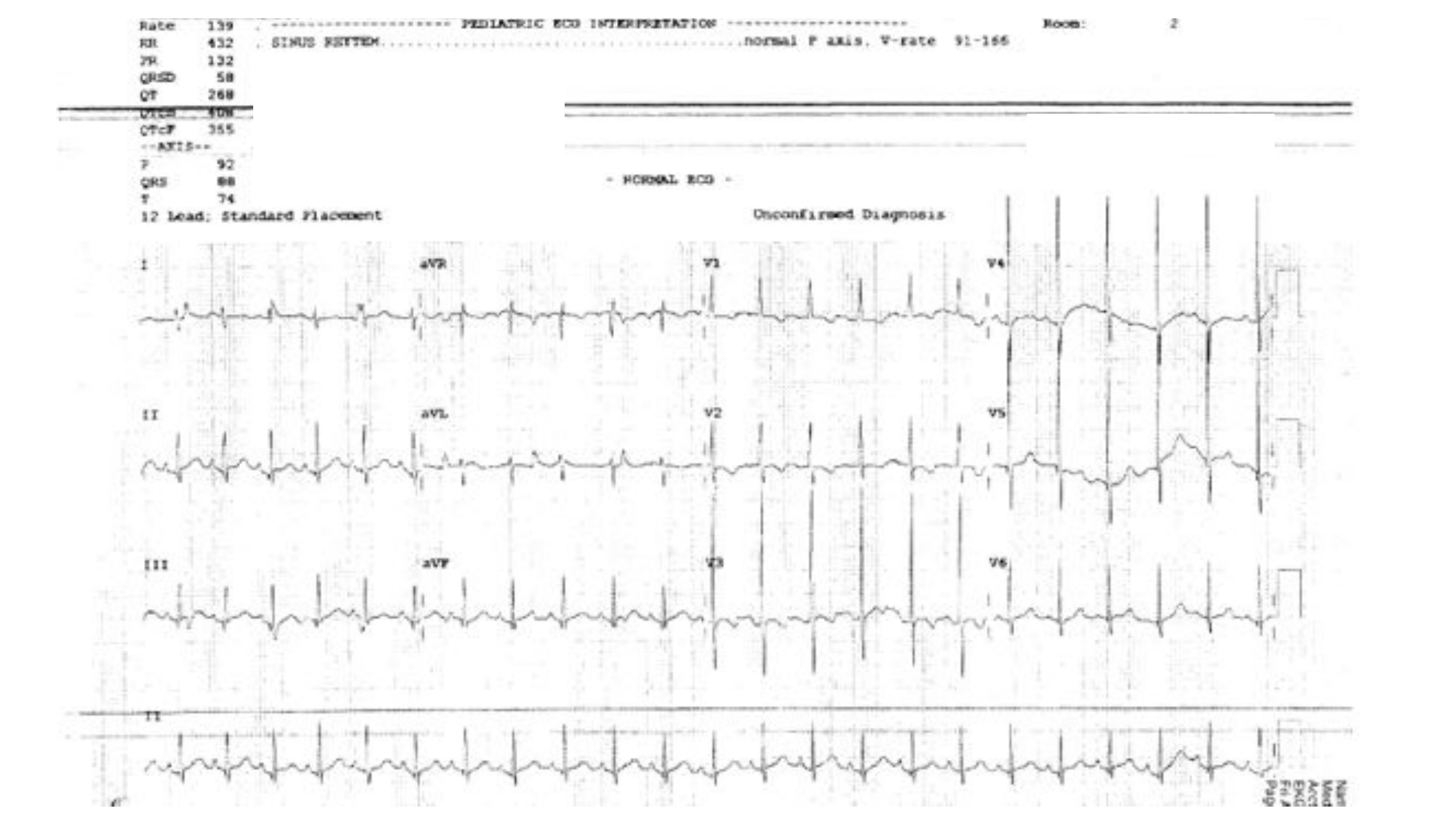

#

## Slide 2
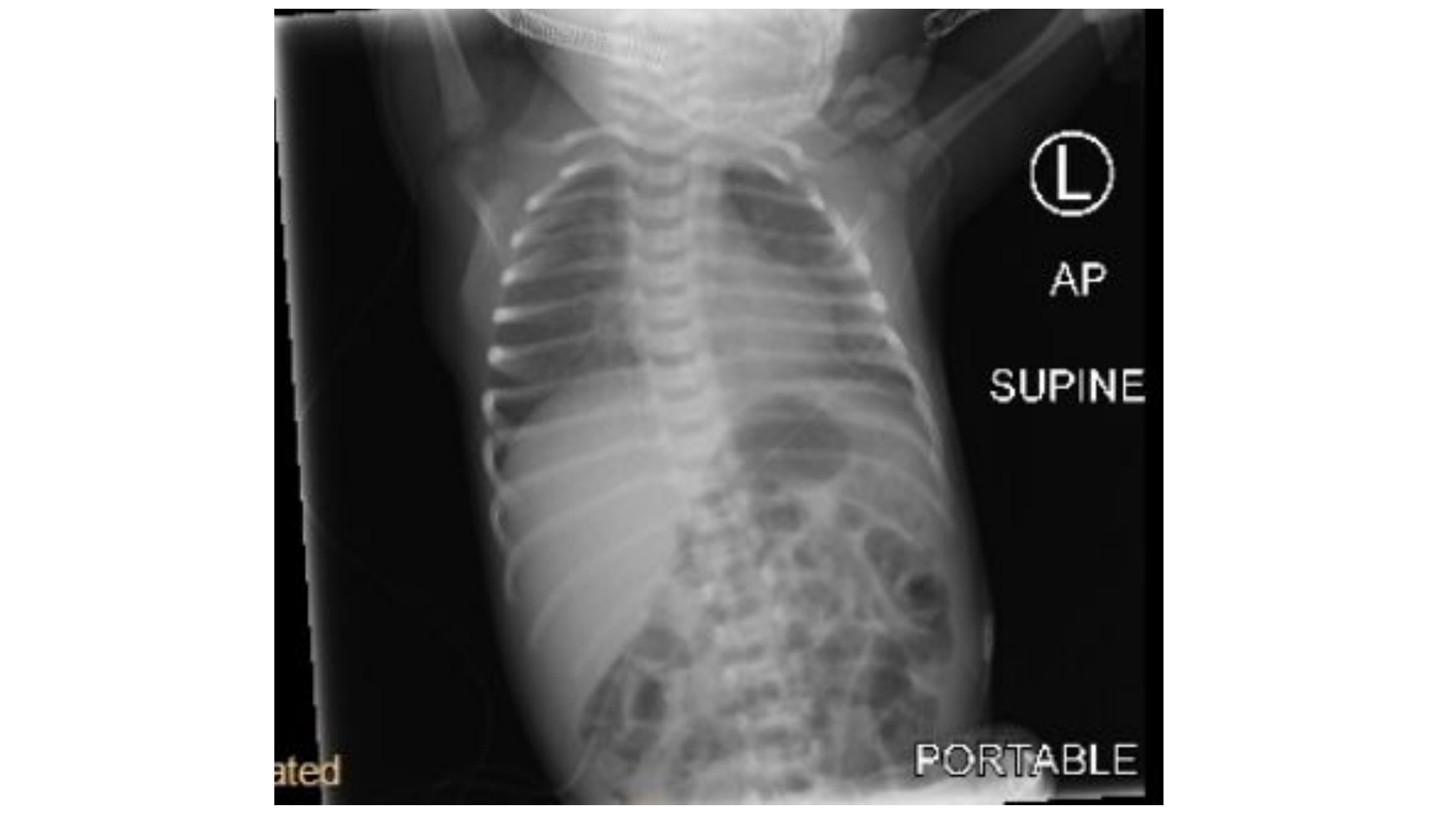

#

Supplement: Supplementary file 1 — Simulation Case.docxEnvironment Preparation.docxImages.pptxTeamwork and Communication Glossary.docxDebriefing Guide.docxEvaluation Form.docxDidactics.pptx [file mep_2374-8265.11089-s001.zip › C. Images.pptx]
